# The Influence of Acute Beta-Hydroxy Beta-Methylbutyrate (HMB) Ingestion on the Human Skeletal Muscle Transcriptome

**DOI:** 10.3390/nu18030434

**Published:** 2026-01-28

**Authors:** Daniel J. Wilkinson, Iain J. Gallagher, Hannah Crossland, Suzette L. Pereira, Ricardo Rueda, Bethan E. Phillips, Kenneth Smith, Colleen S. Deane, Philip J. Atherton

**Affiliations:** 1 Centre of Metabolism, Ageing & Physiology, National Institute of Health Research (NIHR) Biomedical Research Centre (BRC), University of Nottingham, Nottingham NG7 2RD, UK; daniel.wilkinson@nottingham.ac.uk (D.J.W.); hannah.crossland2@nottingham.ac.uk (H.C.); beth.phillips@nottingham.ac.uk (B.E.P.); ken.smith@nottingham.ac.uk (K.S.); 2 Centre for Biomedicine & Global Health, Napier University, Edinburgh EH11 4BN, UK; i.gallagher@napier.ac.uk; 3 Abbott Nutrition, Columbus, OH 43219, USA; suzette.pereira@abbott.com; 4 Abbott Nutrition, 18004 Granada, Spain; ricardo.rueda@abbott.com; 5 Human Development & Health, Faculty of Medicine, University of Southampton, Southampton General Hospital, Southampton SO16 6YD, UK; 6 National Institute of Health Research (NIHR) Southampton Biomedical Research Centre (BRC), University Hospital Southampton NHS Foundation Trust and University of Southampton, Southampton SO16 6YD, UK; 7 Faculty of Sport and Health Science, Ritsumeikan Advanced Research Academy (RARA), Ritsumeikan University, Kyoto 525-8577, Japan

**Keywords:** skeletal muscle, beta-hydroxy beta-methylbutyrate (HMB), transcriptome, RNA sequencing, circadian rhythm, inflammation, amino acid transporters

## Abstract

**Background**: Nutritional interventions to mitigate age/disease-related skeletal muscle attrition are much needed given the growing older population. Beta-hydroxy beta-methylbutyrate (HMB), an endogenous metabolite of the essential amino acid leucine, has anabolic properties in skeletal muscle: acutely stimulating muscle protein synthesis and attenuating muscle protein breakdown. While the role of supplemental HMB on muscle protein turnover is established, mechanistic effects on the muscle transcriptome have not been examined. **Methods**: Total RNA was extracted from m. vastus lateralis muscle biopsies of young males (*n* = 14) before and ~2.5 h after oral consumption of ~3 g HMB. Global changes in the muscle transcriptome were assessed via RNA sequencing, and differential expression in genes between fasted and ‘fed’ (HMB) conditions was determined. To identify the functional biology of differentially expressed genes, gene set enrichment and active subnetwork-orientated enrichment analyses was performed. **Results**: Of 15,982 genes detected, 468 were significantly upregulated and 326 were significantly downregulated in response to HMB. These genes were found to be associated with molecular pathways regulating muscle protein turnover, most notably, JAK-STAT signalling (e.g., *STAM*), circadian rhythm (e.g., *NR1D1*, *NR1D2*, *PER2*, *PER3*), TNFα signalling (e.g., *TNFRSF1A*, *CCL2*, *CXCL2*), and protein synthesis (e.g., *POLR1A*, *POLR2A*, *POLR3A*, *PIK3RR*, *SGK1*). HMB also regulated the expression of AA transporters, evoking a robust increase in *SLC36A1* (*PAT1*) and *SLC7A5* (*LAT1*). **Conclusions**: HMB evokes transcriptional events important in the homeostasis of muscle, supporting a role in proteostasis and one akin to protein intake, i.e., upregulation of AA transporters. Future work should further define HMB’s transcriptomic/proteomic effects in ageing/disease and synergy with exercise.

## 1. Introduction

The post-prandial stimulation of muscle protein synthesis (MPS) in response to nutrition is obligatory for the maintenance of skeletal muscle mass on a long-term basis [1,2,3]. This is exemplified by muscle wasting in states of sustained caloric deficit, e.g., fasting or malnutrition or states of impaired “anabolic resistance” such as ageing [4] or immobilisation [5,6]. Increases in MPS are driven by the protein constituents of food [7]. Upon digestion/absorption, derivative short peptides and amino acids (AAs) are transported to tissues, such as skeletal muscle, enter the cells via specialized AA transporters, and upregulate activity of the mRNA translational machinery. In humans, this acute stimulation of MPS is a transient (only lasting ~2–3 h) but crucial response for muscle maintenance [1,7].

A significant body of prior work has shown that of all proteinogenic AAs, leucine is the most potent in driving the anabolic response to dietary protein [8,9]. Comprising ~8% of high-quality protein, leucine has evolved as a signal as well as a substrate for the stimulation of MPS. Mechanistically, stimulation involves activation of the mechanistic target of rapamycin (mTOR) pathway via GTPases that bind to Raptor and promote lysosomal translocation of mTORC1. Addition of leucine to muscle cell culture medium induces mTOR signalling more powerfully than all other essential AAs (EAAs) [8]. Moreover, provision of just ~3 g L-leucine alone to humans stimulates MPS in the absence of additional EAAs [9].

Leucine, as a branched-chain AA, is also capable of undergoing downstream metabolism within muscle. Briefly, leucine is deaminated by branched-chain amino acid transferase (BCAT) with subsequent irreversible decarboxylation by branched-chain keto-acid dehydrogenase [9]. The formation of downstream metabolites including isovaleryl CoA, and beta-hydroxy beta-methylbutyrate (HMB) then occurs prior to formation of acetyl CoA and acetoacetate, which may feed into ATP production via the tricarboxylic acid (TCA) cycle [9]. The unique signalling properties of leucine in regulating MPS responses to protein nutrition [8] has led to speculation that the formation of leucine metabolites may be in part responsible. Of the leucine metabolites formed, HMB has received most attention since its discovery owing to its use as an ergogenic metabolite in sports and inclusion in oral nutritional supplements in clinical settings [10,11].

Reflecting this, we showed in humans that HMB has similar potency to leucine in stimulating MPS, while simultaneously reducing muscle protein breakdown (MPB) [9]. Prior work has shown how dietary protein [12,13], EAAs [14], and the hormone insulin [15] impact skeletal muscle gene expression, illuminating the mechanisms associated with the nutritional regulation of MPS. To generate an improved understanding of mechanisms of action of HMB, we conducted a transcriptomic analysis of human skeletal muscle samples using RNA sequencing, following consumption of HMB.

## 2. Materials and Methods

### 2.1. Ethical Approval and Study Design

Healthy young males (*n* = 14) were recruited to undertake a feeding study to assess the effects of acute oral administration of HMB on skeletal muscle metabolism. The data presented in this paper represent secondary analyses of muscle samples obtained in previously published work, and volunteer characteristics and previous results are presented in these papers [9,16]. All studies were conducted in accordance with the Declaration of Helsinki, with ethical approval obtained from the University of Nottingham Ethics Committee (B/10/2010). Volunteers were recruited from the local Derbyshire area via poster advertisement. Following recruitment and before inclusion in the project, all volunteers were screened by a physician to exclude for any metabolic, respiratory, cardiovascular/vascular, or other symptoms of ill health. All volunteers provided written informed consent before participation in the study.

Volunteers were asked to refrain from heavy exercise for the 72 h before the study. On the morning of the study (08:30 h), following an overnight fast, volunteers underwent a standardised acute fasted–fed protocol, during which assessments of muscle metabolism were assessed as presented previously [9,16]. Following the fasted period, at the start of which a skeletal muscle biopsy was obtained (fasted), the volunteers consumed 3.42 g of HMB (either calcium salt (flavoured powder) or free acid liquid form (buffered and flavoured) dissolved in ∼100 mL of water (Metabolic Technologies, Inc., Ames, IA, USA)), and 2.5 h later (11 a.m.), another skeletal muscle biopsy was obtained (fed). Muscle biopsies (∼150 mg) were taken from the mid vastus lateralis under sterile conditions using a local anaesthetic (1% lidocaine) and the conchotome technique [17]. While two different forms of HMB (calcium HMB and free acid HMB) were consumed in the two studies being analysed herein, due to the lack of difference in key primary responses (MPS and plasma HMB concentration) to either form, as presented previously [9,16], it was deemed appropriate to combine the two data sources together. Of the *n* = 14 herein, *n* = 7 had consumed calcium HMB and *n* = 7 had consumed free acid HMB.

### 2.2. RNA Extraction and Generation of RNA Sequencing Data

Total RNA was extracted from frozen muscle tissue using TRIzol reagent, and samples with sufficient RNA integrity (RIN ≥ 5.7; all samples) were sequenced using the Illumina HiSeq 3000/HiSeq 4000 platforms (Beijing Genomics Institute, Beijing, China). All raw reads were of sufficient quality (established using FastQC; Babraham Bioinformatics, version 0.11.9). Reads were aligned to the GRCh38 (Ensembl release 96) human genome, and feature counts were generated using the Rsubread package (version 2.13.5) in R (version 4.3.0) [18]. The count data was filtered as recommended by the authors of edgeR (version 3.14) [19], and subsequent normalisation was carried out using the trimmed mean of M values method [20]. A multidimensional scaling (MDS) plot to characterise the variation between samples was conducted using the limma package (version 3.55.5) [21] and is shown in Supplementary Figure S1.

### 2.3. Differential Expression and Gene Category Enrichment

Following normalisation and filtering of the data, differential expression between fasted and fed states was performed using a quasi-likelihood approach and the glmQLFit function of edgeR with a design matrix to account for biological pairing. The quasi-likelihood approach has been recommended by the edgeR developers as having improved false-discovery rate (FDR) control over the standard glmFit method [19,22]. Genes with a Benjamini–Hochberg-corrected *p*-value < 0.05 were defined as differentially expressed. The context of the differentially expressed genes was examined using the pathfindR package (version 2.0.0) [23]. The pathfindR algorithm incorporates protein interaction network (PIN) information when calculating gene category enrichment results. The pathfinder algorithm was run for 25 iterations. Notably, the pathfindR algorithm ranks enriched terms by lowest *p*-value achieved across iterations for plotting purposes. Since this can present an over-optimistic picture of enrichment, the “enrichment_term” function in the pathfindR package was adapted to instead use the highest *p*-value across iterations. See the Supplementary Material R script (enrichment_chart_high_p.R) for full details. The full analysis pathway is described in the diff_exp_analysis_HMBPlusPathfindR.qmd file included in the Supplementary Material.

## 3. Results

### 3.1. Gene Expression Changes Due to Acute Oral Administration of HMB

**Figure 1:**
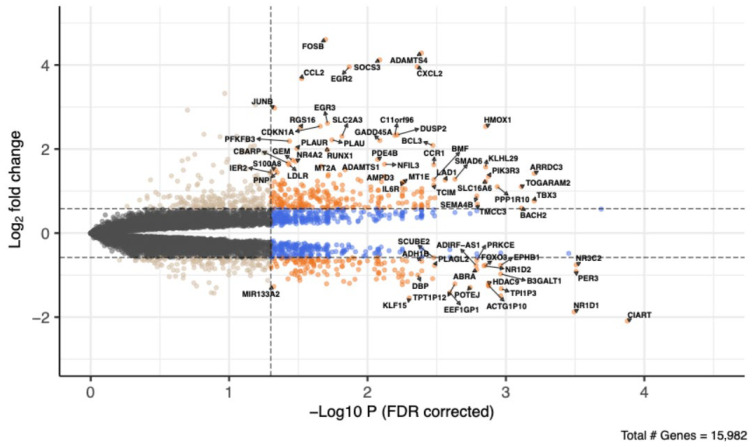
Volcano plot showing the pattern of skeletal muscle gene differential expression between fasted and fed (HMB) conditions following acute oral consumption of 3.42 g of HMB. The vertical dashed line shows a −log_10_ *p*-value cutoff of 1.3 (0.05 on the linear scale). Orange genes have a fold change > |1.3| and an FDR-corrected *p*-value < 0.05. Blue genes have an FDR-corrected *p*-value < 0.05. Selected genes are labelled.

Following normalisation and filtering, there were data available for a total of 15,982 genes. Using the edgeR quasi-likelihood approach, 468 genes were identified as significantly upregulated in expression due to acute HMB feeding, and 326 genes were significantly downregulated (Figure 1).

### 3.2. Category Enrichment Analyses of HMB-Associated Gene Expression Changes

**Figure 2:**
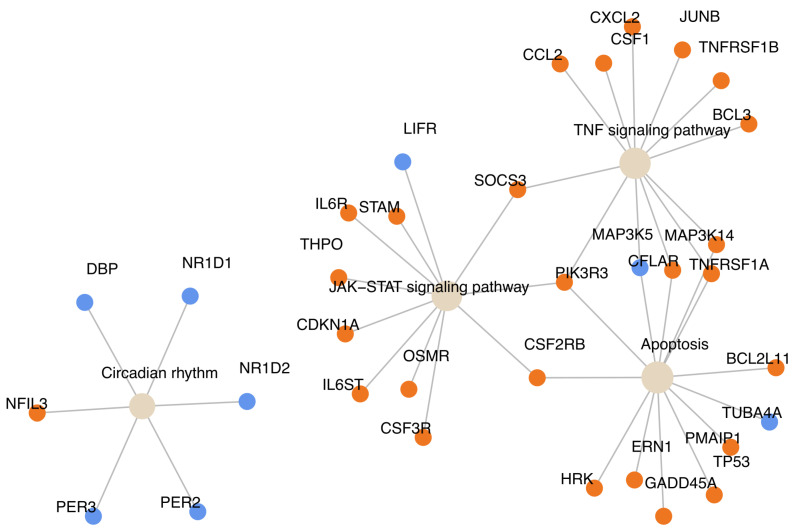
Gene network plot showing selected functionally enriched terms and their associated significantly regulated genes following HMB feeding. Orange represents upregulation, and blue represents downregulation in fed vs. fasted samples.

Next, we examined the transcriptomic context of acute HMB feeding using the pathfindR package [22]. Within the top 20 enriched terms were several pathways known to have key roles in muscle protein turnover, such as TNFα signalling (e.g., *TNFRSF1A*, *CCL2*, *CXCL2*), apoptosis (e.g., *BCL2L11*), MAPK (e.g., *MAP3K14*), and JAK-STAT signalling (e.g., *STAM*) (Figure 2, Supplementary Figures S2–S4, Supplementary File S1). We also observed a robust regulation in genes related to circadian rhythms (upregulation of *NFIL3* and a downregulation of *DBP*, *PER2*, *PER3*, *NR1D1*, and *NR1D2*), RNA polymerase (e.g., *POLR2A*, *POLR1A*, *POLR3A*), and mTOR signalling (e.g., *PIK3RR*, *BRAF*, *SGK1*) (Figure 2, Supplementary Figure S4, Supplementary File S1).

**Figure 3:**
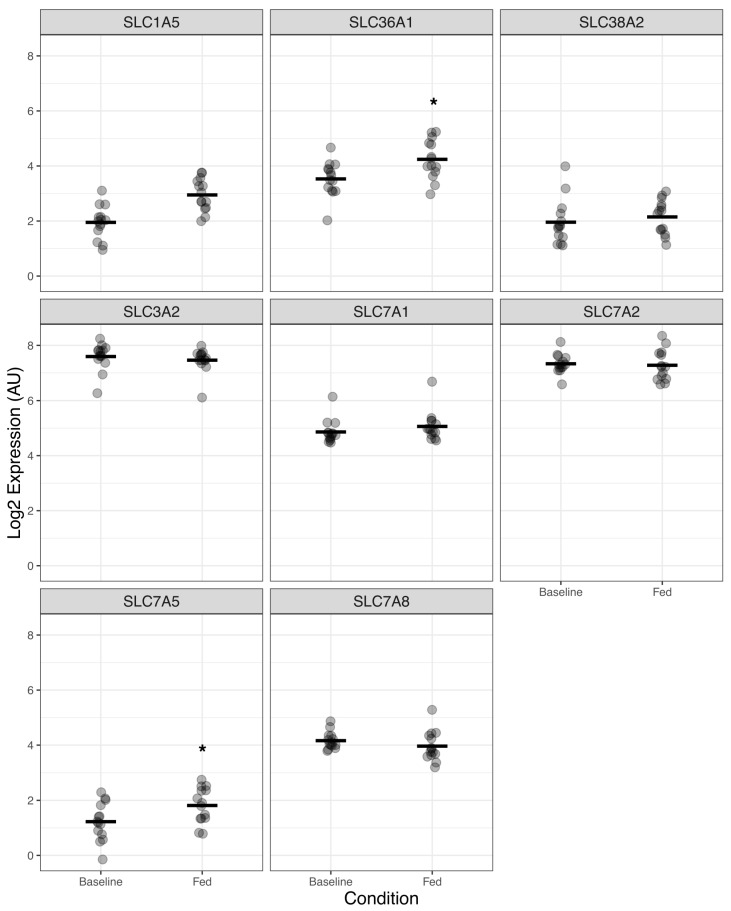
Dotplots showing the impact of acute oral consumption of 3.42 g of HMB on a targeted panel of AA transporters known to regulate the transport of key EAAs across the cell membrane (*n* = 14). * denotes significant difference from baseline.

Finally, based on the known acute pro-anabolic properties of HMB in skeletal muscle [9,16], we took a more targeted approach investigating the transcriptional response of AA transporter genes known to regulate the transport EAAs across the skeletal muscle cell membrane. Across the eight AA transporters investigated, we found that acute HMB feeding upregulated the expression of *SLC36A1* (FDR = 0.004) and *SLC7A5* (FDR = 0.04) (Figure 3, Supplementary File S1).

## 4. Discussion

We investigated the mechanistic effects of HMB on the transcriptome of human skeletal muscle based on prior evidence of its bioavailability in plasma and muscle, the documented anabolic effects on muscle protein turnover [9,16], and the use of HMB in sports science and clinical nutrition [10,11]. Our data demonstrate that HMB exerts substantial transcriptional effects, with robust regulation of inflammatory, protein metabolism, circadian rhythm, and AA transporter genes and pathways. These transcriptional responses are independent of insulin, since HMB does not provoke insulin secretion in the absence of adjuvant macronutrients [15]. While there are few parallels to draw, other studies have investigated the impact of nutrition generally on the muscle transcriptome. A hyper-insulinemic clamp study identified 762 significantly regulated genes (478 up- and 284 downregulated) [15], while studies investigating the impacts of varying protein intake in young adult males also reported a similar number of differentially expressed genes (~300–400 up/downregulated) [12]. Thus, from the limited nutri-omic literature, we can conclude that HMB exerts similar global transcriptional responses to known trophic nutritional factors (i.e., insulin/protein).

In line with HMB’s anabolic role in stimulating MPS [9,16], we observed increases in gene expression of RNA polymerase subunits (e.g., *POLR2A*, *POLR1A*, *POLR3A*) and of genes involved in mTOR-related protein synthesis (e.g., *PIK3RR*, *BRAF*, *SGK1*). Interestingly, *SGK1* has been reported to also increase in response to exercise and to play a role in maintaining muscle homeostasis via downregulating muscle atrophy signalling pathways [24].

Surprisingly, we observed an increase in the expression of “inflammatory-related” genes and pathways in response to acute HMB ingestion, including TNFα signalling (e.g., *TNFRSF1A*, *TNFRSF1B*), NF-κB signalling, and apoptosis. While this gene signature resembles canonical TNFα-NF-κB signalling linked to protein degradation [25], at the physiological level, we observed a decrease in MPB in response to HMB [9,16]. Such divergence between the transcriptional activation and physiological outcome (i.e., MPB) suggests that TNFα–NF-κB signalling could have a context-dependent role, whereby the inflammatory signature may reflect an acute, adaptive remodelling response rather than a pro-catabolic one. Indeed, transient activation of TNFα-NF-κB signalling and other inflammatory mediators is a hallmark of exercise-induced muscle remodelling [26,27], whereby early pro-inflammatory signals facilitate macrophage recruitment, satellite cell activation, and muscle repair [28]. Similarly, TNFα-NF-κB activation has been shown following exposure to high concentrations of branched-chain AAs, albeit not in muscle tissue [29]. Therefore, the observed “pro-inflammatory” gene signature following HMB may represent a controlled response indicative of muscle adaptation. Although speculative, this interpretation aligns with broader evidence that acute pro-inflammatory responses facilitate muscle regeneration and growth. In line with this, HMB has been previously shown to stimulate satellite cell proliferation in both young and aged muscle [30]. Previous chronic supplementation studies have also demonstrated an anti-catabolic effect of HMB via downregulation of the pro-inflammatory genes and pathways including TNF-α, NFκB, and FOXO [31,32,33,34]. In this acute supplementation study, we did observe downregulation of *FOXO3*, typically involved in muscle atrophy signalling cascade [35], while we did not detect a change in expression of *MuRF1* (*TRIM63*), a ubiquitin ligase involved in the ubiquitin–proteasome proteolytic system [36]. It is also worth noting that the volunteers were overnight-fasted and only fed 3.42 g of HMB the following day, so although HMB does boost muscle anabolism, volunteers may still be in a catabolic state due to limited nutritional intake, which may contribute to the observed inflammatory signature.

Skeletal muscle metabolism, especially protein metabolism, is strongly influenced by circadian rhythms, which co-ordinate energy utilisation, metabolic flexibility, and protein turnover across the day [37]. Disruption or modulation of these rhythms can alter nutrient utilisation and protein handling, leading to altered whole-body energy metabolism, muscle weakness, and loss of muscle mass [37]. Within skeletal muscle, each nucleus contains its own molecular clock, which generates circadian rhythms through a transcriptional-translational feedback loop (TTFL), thereby synchronising metabolic processes that underpin whole-body energy homeostasis [37] (for a detailed review of muscle clocks, see [38]). Herein, in response to acute HMB ingestion, we observed a robust regulation of skeletal muscle clock genes, whereby *NFIL3* was upregulated and *DBP*, *PER2*, *PER3*, *NR1D1*, and *NR1D2* were downregulated. Mechanistically, NR1D1 and NR1D2 repress *BMAL1* expression, and PERs supress the transcriptional activity of the BMAL1–CLOCK heterodimer [38]; thus, the downregulation of both (*PERs*, *NR1D1*, and *NR1D2*) due to HMB ingestion may favour BMAL1 activity, which is tightly linked to translational capacity and MPS in muscle [39]. Although the direct metabolic consequences of this acute transcriptional response remain unclear (i.e., in terms of MPS/MPB), circadian rhythms are known to modulate mTORC1 signalling oscillations [40,41]. We therefore hypothesise that the observed clock gene changes reflect a transient drive of muscle clocks to maximise anabolic responsiveness to nutrient intake, contributing to diurnal muscle mass homeostasis. Supporting this, evidence shows *PER2* supresses mTORC1 activity and *PER2* inhibition leads to enhanced protein synthesis [42] and muscle mass preservation [43], implicating *PER2* as a negative regulator of mTOR signalling. Thus, while our data suggests a close bi-directional relationship between nutrition, skeletal muscle clocks, and ultimately protein metabolism, whether acute nutrition (AAs/HMB) does indeed acutely drive muscle clocks in favour of anabolism requires further investigation [44].

AA transporters play a critical role in regulating the availability of intracellular AAs, which serve both as substrates and signals for MPS [3]. The expression and activity of AA transporters is tightly linked to nutrient sensing and mTORC1 signalling, placing them as potentially key modulators of the anabolic responses to feeding [45]. We have previously shown that acute HMB ingestion can increase MPS via increased mTORC1 signalling [9,16] and is thus a potent signal for muscle anabolism. This led us to selectively investigate the impact of HMB on AA transporter genes that are acutely responsive to dietary AAs and are purported to regulate AA transport and the ensuing protein synthetic response [46,47]. We observed a robust increase in *SLC36A1* expression (also known as *PAT1*), an AA transporter located on the lysosomal membrane and essential mediator of AA-dependent mTORC1 activation [48]. Supporting our findings, in vitro leucine treatment increased *SLC36A1* (*PAT1*) expression, leading to mTORC1 activation in the C2C12 cell line [49]. Similarly, whey protein feeding in humans increased *SLC36A1* (*PAT1*) expression concomitant with increased MPS and mTORC1 signalling [50]. We also observed an increase in *SLC7A5* (also known as *LAT1*) (as has been observed in other tissues [51]), the most highly expressed large neutral amino acid transporter in skeletal muscle [52] known for mediating the influx of EAAs into skeletal muscle [53]. Supporting our findings, others have shown increased muscle *LAT1* gene expression and enhanced mTORC1 signalling 1–3 h post EAA ingestion in young adults [46], suggesting that increased *SLC7A5* (*LAT1*) gene expression is a unique regulatory mechanism associated with the muscle protein anabolic response following increased EAA availability. Why and how HMB increases AA transporter gene expression and how this links to increases in MPS remain unknown, but given HMB enters muscle cells via H^+^-coupled monocarboxylate transporters (MCT 1 and 4) and Na^+^-coupled monocarboxylate transporters (SMCT1) [54], and not AA transporters, the effects of HMB on AA transporters are most likely indirect. A possible mechanistic pathway is that HMB enters the muscle cell via MCT1/MCT4/SMCT1 and stimulates mTOR signalling, thereby enhancing the activity of transcription factors that control the expression of AA transporters (e.g., c-Myc controls the expression of *SLC7A5*) [55]. This upregulation in AA transporters may then support increases in MPS via the transport of circulating AAs into the cell to act as substrates and signals for MPS. However, this proposed mechanism remains to be verified.

A potential limitation to our study is that we combined volunteer samples from two independent cohorts consuming either calcium or free acid HMB. However, physiologically, we observed no differences in the key primary responses (MPS and plasma HMB concentration) to either form of HMB (as presented previously [9,16]); thus, we deemed it appropriate to combine the two sample sets to increase statistical power. Nonetheless, larger trials are warranted to confirm our findings. In addition, due to the technical limitation on obtaining multiple muscle biopsies during the 2.5 h timeframe between fasted and fed sampling, we may have missed early transient gene expression signals that may have occurred in response to HMB. Additional studies with more frequent sequential timepoints may help address this limitation. We observed a robust effect of HMB on skeletal muscle clock gene expression; however, this study was not designed to investigate the circadian rhythms insofar as biopsies were not taken repeatedly throughout a 24 h cycle (e.g., constant routine or realistic life-style protocol design [37]) and there were no control (i.e., fasted) samples taken at the 2.5 h sampling timepoint. While we can therefore not rule out that the circadian rhythm gene signature simply reflects the time between biopsies, we strongly believe the muscle clock regulation is due to HMB ingestion for two key reasons: first, the magnitude of change in clock genes was >6-fold, which is significantly more than the ~1.5 fold circadian oscillations reported [56], and second, several of the genes we observed to be significantly downregulated in response to HMB (*PER2*, *PER3*, *DBP*) should have been at peak expression for that time of day [37,56]. Thus, our data strongly point towards a nutri-clock–anabolism axis, which requires further investigation in the form of specifically designed and well-controlled human metabolic and molecular investigations. Another limitation of the present study is the focus on transcriptomic profiling in isolation. While RNA sequencing provides an untargeted approach to investigating gene expression changes in response to HMB supplementation, transcriptional alterations do not necessarily reflect upstream regulatory events, or downstream metabolic consequences. Accordingly, future studies integrating complementary multi-omics approaches, such as epigenomic, proteomic and/or metabolomic analyses alongside transcriptomics, are essential to fully elucidate the regulatory mechanisms underlying HMB-induced molecular changes in skeletal muscle. Finally, as this study was designed to be hypothesis-generating, independent experimental validation of selected differentially expressed genes at the gene and/or protein level (e.g., via qPCR and Western blotting, respectively) was not performed and represents an important focus for future studies.

## 5. Conclusions

In summary, using untargeted transcriptomics, we demonstrate that acute HMB ingestion is sufficient to increase MPS and decrease MPB in young healthy males and elicits a robust transcriptional response, driven largely by changes in genes related to proteostasis, inflammation, and circadian rhythm. While the consequence of these gene signatures remains unknown, we hypothesise that the general upregulation of inflammatory-related genes, genes related to protein synthesis and AA transport, and downregulation in clock-related genes is a transient response to HMB that permits the prioritisation of muscle anabolism for muscle homeostasis. Future work should seek to uncover the mechanistic consequences of these gene signatures and should define HMB’s transcriptional effects in ageing and disease and in the context of exercise.

## Data Availability

The original contributions presented in this study are included in the article.

## Supplementary Materials

The following supporting information can be downloaded at https://www.mdpi.com/article/10.3390/nu18030434/s1, Supplementary Figure S1: Scatterplot of a multidimensional scaling analysis of the transcriptomic data. The plot visualises the difference in expression profiles of the fasted (baseline) and fed (HMB) conditions. Samples are separated by fed (HMB) status in the second dimension. Supplementary Figure S2: Gene network with no selected terms; Supplementary Figure S3: UpSet plot showing how genes are shared across the pathfindR enriched pathways indicated in Figure 1; Supplementary Figure S4: UpSet plot showing how genes are shared across the selected pathfindR enriched pathways indicated in Figure 2; Supplementary File S1; R script: enrichment_chart_high_p.R; Full analysis script: diff_exp_analysis_HMBPlusPathfindR.qmd file.
