# Supplementary material for: The Influence of Acute Beta-Hydroxy Beta-Methylbutyrate (HMB) Ingestion on the Human Skeletal Muscle Transcriptome
Source: Nutrients. 2026 Jan 28;18(3):434. doi: 10.3390/nu18030434 (PMC12899265; doi:10.3390/nu18030434)
Supplement: Supplementary file 1 [file nutrients-18-00434-s001.zip › Supplementary_Figures.pdf]

Supplementary Figures

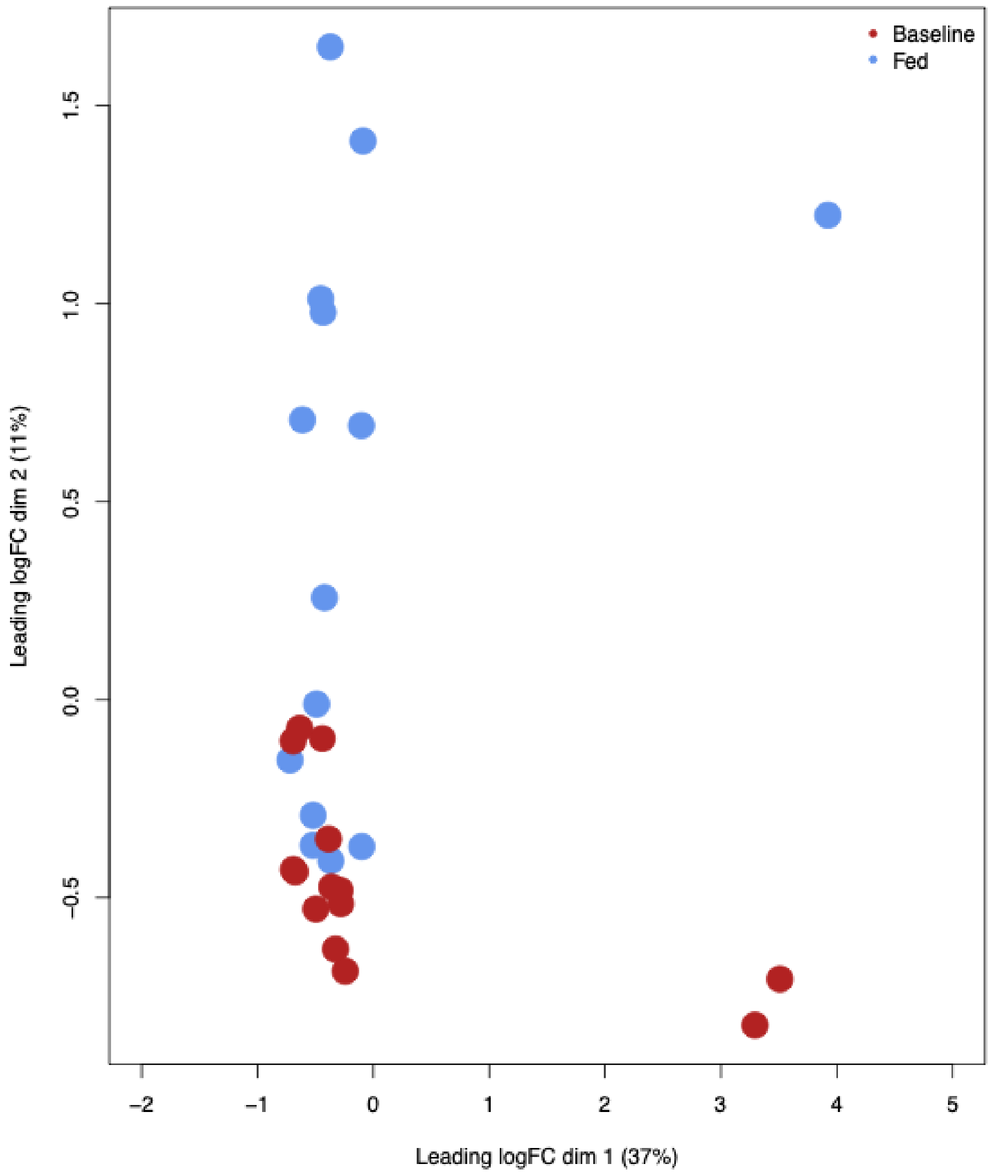

Figure S1: Scatterplot of a multidimensional scaling analysis of the transcriptomic data. The plot visualises the difference in expression profiles of the fasted (Baseline) and fed (HMB) conditions. Samples are separated by fed (HMB) status in the second dimension.

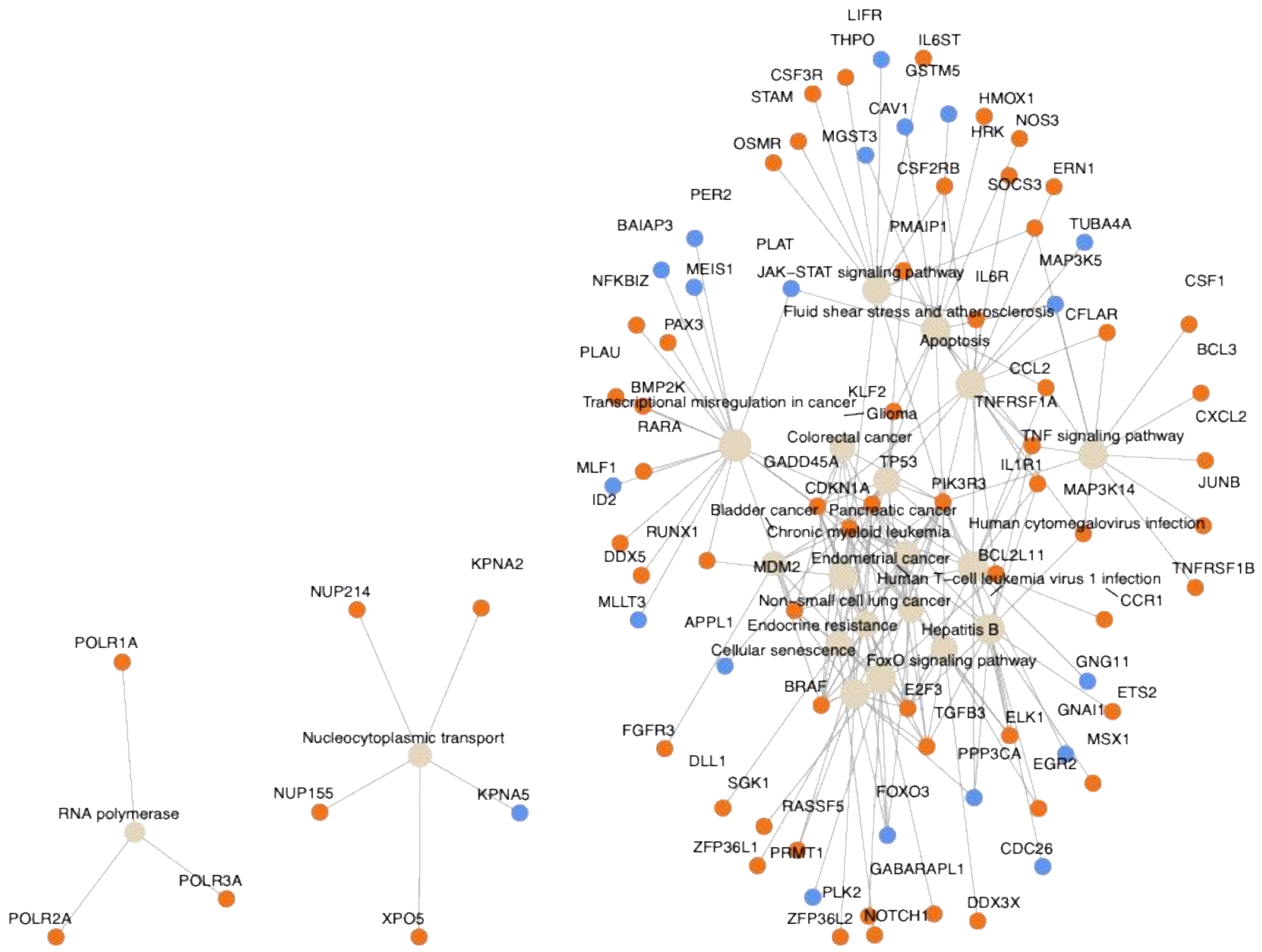

Figure S2: Gene network with no selected terms

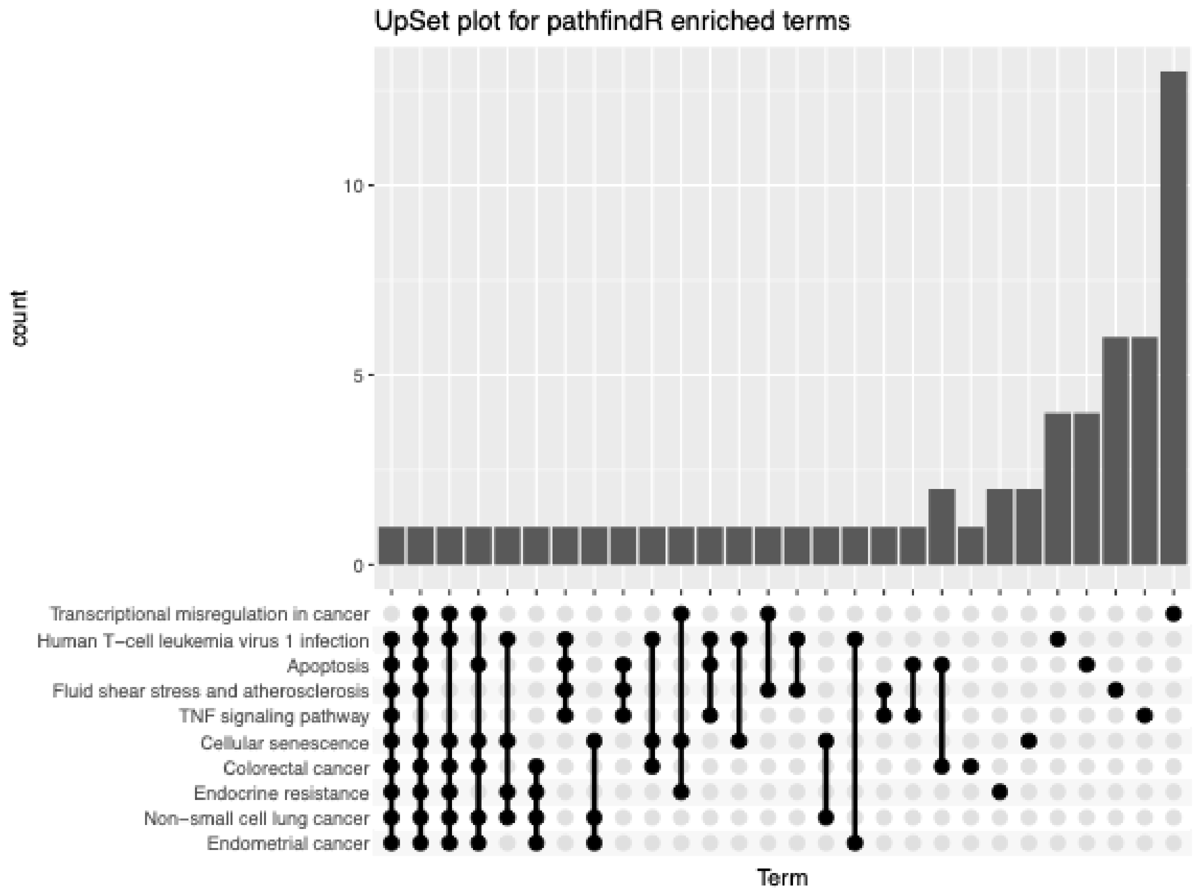

Figure S3: UpSet plot showing how genes are shared across the pathfindR enriched pathways indicated in Figure 1.

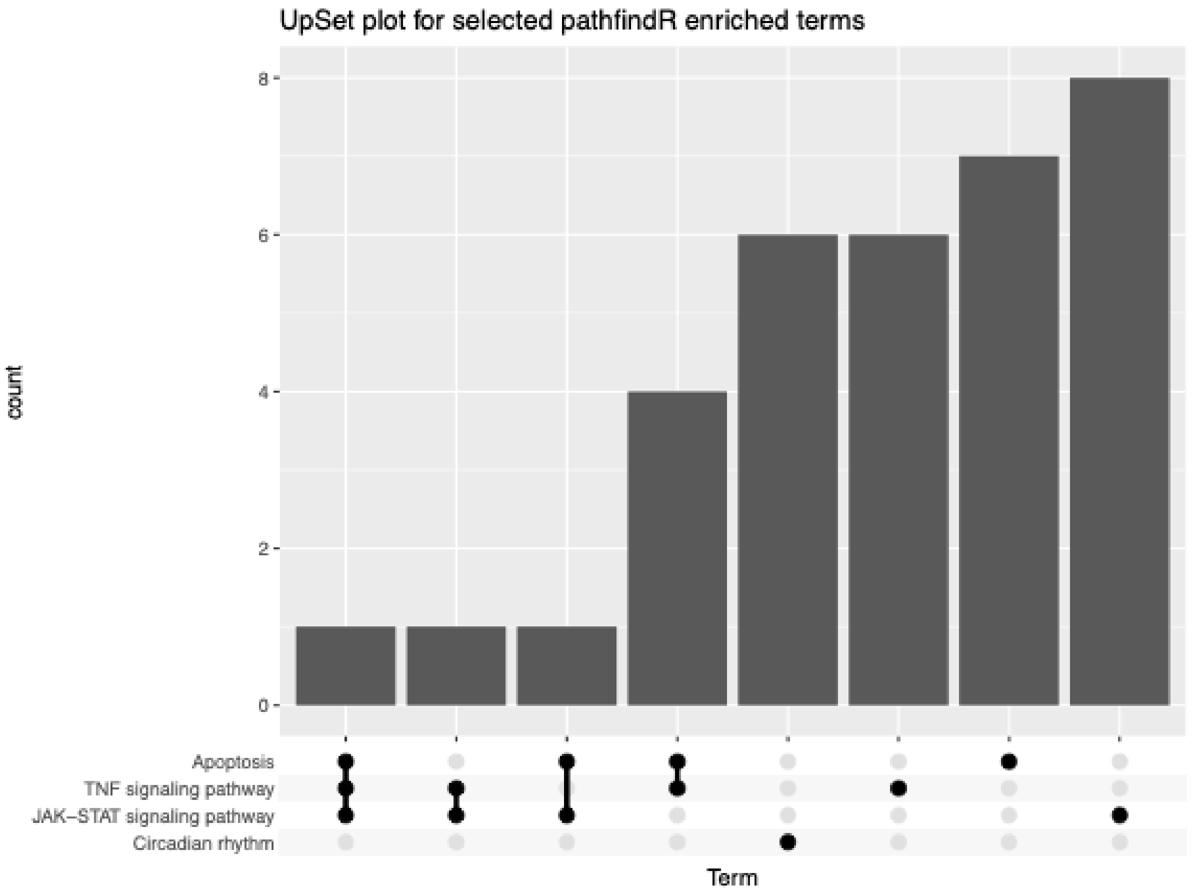

Figure S4: UpSet plot showing how genes are shared across the selected pathfindR enriched pathways indicated in Figure 2.
