# Supplementary material for: The Influence of Acute Beta-Hydroxy Beta-Methylbutyrate (HMB) Ingestion on the Human Skeletal Muscle Transcriptome
Source: Nutrients. 2026 Jan 28;18(3):434. doi: 10.3390/nu18030434 (PMC12899265; doi:10.3390/nu18030434)
Supplement: Supplementary file 1 [file nutrients-18-00434-s001.zip › Supplementary_File_S1.pdf]

Term_Description	genes	ensembl_gene_id	entrezgene_id
TNF signaling pathway	TNFRSF1A	ENSG00000067182	7132
TNF signaling pathway	TNFRSF1B	ENSG00000028137	7133
TNF signaling pathway	PIK3R3	ENSG00000117461	8503
TNF signaling pathway	MAP3K14	ENSG00000006062	9020
TNF signaling pathway	CFLAR	ENSG00000003402	8837
TNF signaling pathway	CCL2	ENSG00000108691	6347
TNF signaling pathway	CXCL2	ENSG00000081041	2920
TNF signaling pathway	CSF1	ENSG00000184371	1435
TNF signaling pathway	BCL3	ENSG00000069399	602
TNF signaling pathway	SOCS3	ENSG00000184557	9021
TNF signaling pathway	MAP3K5	ENSG00000197442	4217
Apoptosis	TP53	ENSG00000141510	7157
Apoptosis	MAP3K14	ENSG00000006062	9020
Apoptosis	CFLAR	ENSG00000003402	8837
Apoptosis	PIK3R3	ENSG00000117461	8503
Apoptosis	CSF2RB	ENSG00000100368	1439
Apoptosis	TNFRSF1A	ENSG00000067182	7132
Apoptosis	PMAIP1	ENSG00000141682	5366
Apoptosis	ERN1	ENSG00000178607	2081
Apoptosis	GADD45A	ENSG00000116717	1647
Apoptosis	BCL2L11	ENSG00000153094	10018
Apoptosis	MAP3K5	ENSG00000197442	4217
Apoptosis	TUBA4A	ENSG00000127824	7277
Endometrial cancer	ELK1	ENSG00000126767	2002
Endometrial cancer	PIK3R3	ENSG00000117461	8503
Endometrial cancer	BRAF	ENSG00000157764	673
Endometrial cancer	TP53	ENSG00000141510	7157
Endometrial cancer	CDKN1A	ENSG00000124762	1026
Endometrial cancer	GADD45A	ENSG00000116717	1647
Endometrial cancer	FOXO3	ENSG00000118689	2309
Human T-cell leukemia virus 1	TGFB3	ENSG00000119699	7043
Human T-cell leukemia virus 1	CDKN1A	ENSG00000124762	1026
Human T-cell leukemia virus 1	MAP3K14	ENSG00000006062	9020
Human T-cell leukemia virus 1	TNFRSF1A	ENSG00000067182	7132
Human T-cell leukemia virus 1	IL1R1	ENSG00000115594	3554
Human T-cell leukemia virus 1	PIK3R3	ENSG00000117461	8503
Human T-cell leukemia virus 1	E2F3	ENSG00000112242	1871
Human T-cell leukemia virus 1	ELK1	ENSG00000126767	2002
Human T-cell leukemia virus 1	ETS2	ENSG00000157557	2114
Human T-cell leukemia virus 1	EGR2	ENSG00000122877	1959
Human T-cell leukemia virus 1	MSX1	ENSG00000163132	4487
Human T-cell leukemia virus 1	TP53	ENSG00000141510	7157
Human T-cell leukemia virus 1	PPP3CA	ENSG00000138814	5530
Human T-cell leukemia virus 1	CDC26	ENSG00000176386	246184
Fluid shear stress and atherosc	PIK3R3	ENSG00000117461	8503
Fluid shear stress and atherosc	CCL2	ENSG00000108691	6347
Fluid shear stress and atherosc	KLF2	ENSG00000127528	10365
Fluid shear stress and atherosc	HMOX1	ENSG00000100292	3162
Fluid shear stress and atherosc	NOS3	ENSG00000164867	4846
Fluid shear stress and atherosc	TP53	ENSG00000141510	7157
Fluid shear stress and atherosc	TNFRSF1A	ENSG00000067182	7132
Fluid shear stress and atherosc	IL1R1	ENSG00000115594	3554
Fluid shear stress and atherosc	GSTM5	ENSG00000134201	2949
Fluid shear stress and atherosc	MGST3	ENSG00000143198	4259
Fluid shear stress and atherosc	PLAT	ENSG00000104368	5327
Fluid shear stress and atherosc	MAP3K5	ENSG00000197442	4217
Fluid shear stress and atherosc	CAV1	ENSG00000105974	857
JAK-STAT signaling pathway	THPO	ENSG00000090534	7066

JAK-STAT signaling pathway	IL6R	ENSG00000160712	3570
JAK-STAT signaling pathway	IL6ST	ENSG00000134352	3572
JAK-STAT signaling pathway	OSMR	ENSG00000145623	9180
JAK-STAT signaling pathway	SOCS3	ENSG00000184557	9021
JAK-STAT signaling pathway	CDKN1A	ENSG00000124762	1026
JAK-STAT signaling pathway	PIK3R3	ENSG00000117461	8503
JAK-STAT signaling pathway	CSF2RB	ENSG00000100368	1439
JAK-STAT signaling pathway	STAM	ENSG00000136738	8027
JAK-STAT signaling pathway	LIFR	ENSG00000113594	3977
Hepatitis B	BRAF	ENSG00000157764	673
Hepatitis B	ELK1	ENSG00000126767	2002
Hepatitis B	PIK3R3	ENSG00000117461	8503
Hepatitis B	TGFB3	ENSG00000119699	7043
Hepatitis B	CDKN1A	ENSG00000124762	1026
Hepatitis B	EGR2	ENSG00000122877	1959
Hepatitis B	TP53	ENSG00000141510	7157
Hepatitis B	DDX3X	ENSG00000215301	1654
Hepatitis B	E2F3	ENSG00000112242	1871
Colorectal cancer	TGFB3	ENSG00000119699	7043
Colorectal cancer	PIK3R3	ENSG00000117461	8503
Colorectal cancer	BRAF	ENSG00000157764	673
Colorectal cancer	TP53	ENSG00000141510	7157
Colorectal cancer	CDKN1A	ENSG00000124762	1026
Colorectal cancer	GADD45A	ENSG00000116717	1647
Colorectal cancer	PMAIP1	ENSG00000141682	5366
Colorectal cancer	BCL2L11	ENSG00000153094	10018
Colorectal cancer	APPL1	ENSG00000157500	26060
Transcriptional misregulation in	PAX3	ENSG00000135903	5077
Transcriptional misregulation in	ID2	ENSG00000115738	3398
Transcriptional misregulation in	CDKN1A	ENSG00000124762	1026
Transcriptional misregulation in	NFKBIZ	ENSG00000144802	64332
Transcriptional misregulation in	DDX5	ENSG00000108654	1655
Transcriptional misregulation in	PLAU	ENSG00000122861	5328
Transcriptional misregulation in	RUNX1	ENSG00000159216	861
Transcriptional misregulation in	RARA	ENSG00000131759	5914
Transcriptional misregulation in	BMP2K	ENSG00000138756	55589
Transcriptional misregulation in	TP53	ENSG00000141510	7157
Transcriptional misregulation in	GADD45A	ENSG00000116717	1647
Transcriptional misregulation in	BAIAP3	ENSG00000007516	8938
Transcriptional misregulation in	MLF1	ENSG00000178053	4291
Transcriptional misregulation in	PER2	ENSG00000132326	8864
Transcriptional misregulation in	MLLT3	ENSG00000171843	4300
Transcriptional misregulation in	MEIS1	ENSG00000143995	4211
Transcriptional misregulation in	PLAT	ENSG00000104368	5327
Nucleocytoplasmic transport	XPO5	ENSG00000124571	57510
Nucleocytoplasmic transport	NUP155	ENSG00000113569	9631
Nucleocytoplasmic transport	NUP214	ENSG00000126883	8021
Nucleocytoplasmic transport	KPNA2	ENSG00000182481	3838
Nucleocytoplasmic transport	KPNA5	ENSG00000196911	3841
RNA polymerase	POLR2A	ENSG00000181222	5430
RNA polymerase	POLR1A	ENSG00000068654	25885
RNA polymerase	POLR3A	ENSG00000148606	11128
MAPK signaling pathway	DUSP2	ENSG00000158050	1844
MAPK signaling pathway	MAP3K14	ENSG00000006062	9020
MAPK signaling pathway	BRAF	ENSG00000157764	673
MAPK signaling pathway	TP53	ENSG00000141510	7157
MAPK signaling pathway	ELK1	ENSG00000126767	2002
MAPK signaling pathway	GADD45A	ENSG00000116717	1647
MAPK signaling pathway	IL1R1	ENSG00000115594	3554

MAPK signaling pathway	TNFRSF1A	ENSG00000067182	7132
MAPK signaling pathway	TGFB3	ENSG00000119699	7043
MAPK signaling pathway	CSF1	ENSG00000184371	1435
MAPK signaling pathway	DUSP7	ENSG00000164086	1849
MAPK signaling pathway	CACNG1	ENSG00000108878	786
MAPK signaling pathway	MAP3K5	ENSG00000197442	4217
MAPK signaling pathway	HSPA1L	ENSG00000204390	3305
MAPK signaling pathway	PPP3CA	ENSG00000138814	5530
MAPK signaling pathway	ANGPT1	ENSG00000154188	284
Non-small cell lung cancer	PIK3R3	ENSG00000117461	8503
Non-small cell lung cancer	E2F3	ENSG00000112242	1871
Non-small cell lung cancer	RASSF5	ENSG00000266094	83593
Non-small cell lung cancer	BRAF	ENSG00000157764	673
Non-small cell lung cancer	TP53	ENSG00000141510	7157
Non-small cell lung cancer	CDKN1A	ENSG00000124762	1026
Non-small cell lung cancer	GADD45A	ENSG00000116717	1647
Non-small cell lung cancer	FOXO3	ENSG00000118689	2309
Pancreatic cancer	PIK3R3	ENSG00000117461	8503
Pancreatic cancer	BRAF	ENSG00000157764	673
Pancreatic cancer	E2F3	ENSG00000112242	1871
Pancreatic cancer	TGFB3	ENSG00000119699	7043
Pancreatic cancer	TP53	ENSG00000141510	7157
Pancreatic cancer	CDKN1A	ENSG00000124762	1026
Pancreatic cancer	GADD45A	ENSG00000116717	1647
Cellular senescence	RASSF5	ENSG00000266094	83593
Cellular senescence	TP53	ENSG00000141510	7157
Cellular senescence	PIK3R3	ENSG00000117461	8503
Cellular senescence	CDKN1A	ENSG00000124762	1026
Cellular senescence	E2F3	ENSG00000112242	1871
Cellular senescence	GADD45A	ENSG00000116717	1647
Cellular senescence	ZFP36L1	ENSG00000185650	677
Cellular senescence	ZFP36L2	ENSG00000152518	678
Cellular senescence	TGFB3	ENSG00000119699	7043
Cellular senescence	FOXO3	ENSG00000118689	2309
Cellular senescence	PPP3CA	ENSG00000138814	5530
Kaposi sarcoma-associated he	PIK3R3	ENSG00000117461	8503
Kaposi sarcoma-associated he	TNFRSF1A	ENSG00000067182	7132
Kaposi sarcoma-associated he	IL6ST	ENSG00000134352	3572
Kaposi sarcoma-associated he	CDKN1A	ENSG00000124762	1026
Kaposi sarcoma-associated he	TP53	ENSG00000141510	7157
Kaposi sarcoma-associated he	E2F3	ENSG00000112242	1871
Kaposi sarcoma-associated he	CCR1	ENSG00000163823	1230
Kaposi sarcoma-associated he	MAP1LC3B2	ENSG00000258102	643246
Kaposi sarcoma-associated he	CXCL2	ENSG00000081041	2920
Kaposi sarcoma-associated he	GNG11	ENSG00000127920	2791
Kaposi sarcoma-associated he	PPP3CA	ENSG00000138814	5530
Kaposi sarcoma-associated he	RPS27A	ENSG00000143947	6233
Prostate cancer	CDKN1A	ENSG00000124762	1026
Prostate cancer	E2F3	ENSG00000112242	1871
Prostate cancer	TP53	ENSG00000141510	7157
Prostate cancer	PIK3R3	ENSG00000117461	8503
Prostate cancer	BRAF	ENSG00000157764	673
Prostate cancer	PLAU	ENSG00000122861	5328
Prostate cancer	PLAT	ENSG00000104368	5327
Circadian rhythm	NFIL3	ENSG00000165030	4783
Circadian rhythm	PER3	ENSG00000049246	8863
Circadian rhythm	PER2	ENSG00000132326	8864
Circadian rhythm	DBP	ENSG00000105516	1628
Circadian rhythm	NR1D1	ENSG00000126368	9572

Circadian rhythm	NR1D2	ENSG00000174738	9975
Human cytomegalovirus infecti	PIK3R3	ENSG00000117461	8503
Human cytomegalovirus infecti	TNFRSF1A	ENSG00000067182	7132
Human cytomegalovirus infecti	CCL2	ENSG00000108691	6347
Human cytomegalovirus infecti	IL6R	ENSG00000160712	3570
Human cytomegalovirus infecti	IL1R1	ENSG00000115594	3554
Human cytomegalovirus infecti	CCR1	ENSG00000163823	1230
Human cytomegalovirus infecti	CDKN1A	ENSG00000124762	1026
Human cytomegalovirus infecti	TP53	ENSG00000141510	7157
Human cytomegalovirus infecti	E2F3	ENSG00000112242	1871
Human cytomegalovirus infecti	ELK1	ENSG00000126767	2002
Human cytomegalovirus infecti	GNG11	ENSG00000127920	2791
Human cytomegalovirus infecti	PPP3CA	ENSG00000138814	5530
Human cytomegalovirus infecti	GNAI1	ENSG00000127955	2770
Chronic myeloid leukemia	RUNX1	ENSG00000159216	861
Chronic myeloid leukemia	TP53	ENSG00000141510	7157
Chronic myeloid leukemia	TGFB3	ENSG00000119699	7043
Chronic myeloid leukemia	CDKN1A	ENSG00000124762	1026
Chronic myeloid leukemia	E2F3	ENSG00000112242	1871
Chronic myeloid leukemia	PIK3R3	ENSG00000117461	8503
Chronic myeloid leukemia	BRAF	ENSG00000157764	673
Chronic myeloid leukemia	GADD45A	ENSG00000116717	1647
Hepatitis C	CLDN5	ENSG00000184113	7122
Hepatitis C	SOCS3	ENSG00000184557	9021
Hepatitis C	BRAF	ENSG00000157764	673
Hepatitis C	PIK3R3	ENSG00000117461	8503
Hepatitis C	TP53	ENSG00000141510	7157
Hepatitis C	CDKN1A	ENSG00000124762	1026
Hepatitis C	TNFRSF1A	ENSG00000067182	7132
Hepatitis C	LDLR	ENSG00000130164	3949
Hepatitis C	MX1	ENSG00000157601	4599
Hepatitis C	MX2	ENSG00000183486	4600
Hepatitis C	E2F3	ENSG00000112242	1871
Hepatitis C	CFLAR	ENSG00000003402	8837
Endocrine resistance	BRAF	ENSG00000157764	673
Endocrine resistance	PIK3R3	ENSG00000117461	8503
Endocrine resistance	NOTCH1	ENSG00000148400	4851
Endocrine resistance	TP53	ENSG00000141510	7157
Endocrine resistance	CDKN1A	ENSG00000124762	1026
Endocrine resistance	E2F3	ENSG00000112242	1871
Endocrine resistance	DLL1	ENSG00000198719	28514
Lipid and atherosclerosis	PIK3R3	ENSG00000117461	8503
Lipid and atherosclerosis	TP53	ENSG00000141510	7157
Lipid and atherosclerosis	NOS3	ENSG00000164867	4846
Lipid and atherosclerosis	CCL2	ENSG00000108691	6347
Lipid and atherosclerosis	ABCA1	ENSG00000165029	19
Lipid and atherosclerosis	ERN1	ENSG00000178607	2081
Lipid and atherosclerosis	ATF6	ENSG00000118217	22926
Lipid and atherosclerosis	CXCL2	ENSG00000081041	2920
Lipid and atherosclerosis	TNFRSF1A	ENSG00000067182	7132
Lipid and atherosclerosis	POU2F1	ENSG00000143190	5451
Lipid and atherosclerosis	LDLR	ENSG00000130164	3949
Lipid and atherosclerosis	MAP3K5	ENSG00000197442	4217
Lipid and atherosclerosis	PPP3CA	ENSG00000138814	5530
Lipid and atherosclerosis	HSPA1L	ENSG00000204390	3305
Ferroptosis	TP53	ENSG00000141510	7157
Ferroptosis	MAP1LC3B2	ENSG00000258102	643246
Ferroptosis	HMOX1	ENSG00000100292	3162
Ferroptosis	ACSL1	ENSG00000151726	2180

Ferroptosis	ACSL3	ENSG00000123983	2181
Ferroptosis	LPCAT3	ENSG00000111684	10162
Ferroptosis	ACSL6	ENSG00000164398	23305
Thyroid cancer	BRAF	ENSG00000157764	673
Thyroid cancer	TP53	ENSG00000141510	7157
Thyroid cancer	CDKN1A	ENSG00000124762	1026
Thyroid cancer	GADD45A	ENSG00000116717	1647
Sphingolipid signaling pathway	PIK3R3	ENSG00000117461	8503
Sphingolipid signaling pathway	SPHK2	ENSG00000063176	56848
Sphingolipid signaling pathway	ABCC1	ENSG00000103222	4363
Sphingolipid signaling pathway	TP53	ENSG00000141510	7157
Sphingolipid signaling pathway	NOS3	ENSG00000164867	4846
Sphingolipid signaling pathway	TNFRSF1A	ENSG00000067182	7132
Sphingolipid signaling pathway	GNAI1	ENSG00000127955	2770
Sphingolipid signaling pathway	MAP3K5	ENSG00000197442	4217
Sphingolipid signaling pathway	PRKCE	ENSG00000171132	5581
Neurotrophin signaling pathway	PIK3R3	ENSG00000117461	8503
Neurotrophin signaling pathway	BRAF	ENSG00000157764	673
Neurotrophin signaling pathway	TP53	ENSG00000141510	7157
Neurotrophin signaling pathway	SH2B3	ENSG00000111252	10019
Neurotrophin signaling pathway	ZNF274	ENSG00000171606	10782
Neurotrophin signaling pathway	PSEN1	ENSG00000080815	5663
Neurotrophin signaling pathway	FOXO3	ENSG00000118689	2309
Neurotrophin signaling pathway	MAP3K5	ENSG00000197442	4217
FoxO signaling pathway	BRAF	ENSG00000157764	673
FoxO signaling pathway	PIK3R3	ENSG00000117461	8503
FoxO signaling pathway	CDKN1A	ENSG00000124762	1026
FoxO signaling pathway	PLK2	ENSG00000145632	10769
FoxO signaling pathway	GADD45A	ENSG00000116717	1647
FoxO signaling pathway	BCL2L11	ENSG00000153094	10018
FoxO signaling pathway	KLF2	ENSG00000127528	10365
FoxO signaling pathway	GABARAPL1	ENSG00000139112	23710
FoxO signaling pathway	SGK1	ENSG00000118515	6446
FoxO signaling pathway	TGFB3	ENSG00000119699	7043
FoxO signaling pathway	FOXO3	ENSG00000118689	2309
Breast cancer	NCOA1	ENSG00000084676	8648
Breast cancer	BRAF	ENSG00000157764	673
Breast cancer	NOTCH1	ENSG00000148400	4851
Breast cancer	DLL1	ENSG00000198719	28514
Breast cancer	PIK3R3	ENSG00000117461	8503
Breast cancer	FZD5	ENSG00000163251	7855
Breast cancer	E2F3	ENSG00000112242	1871
Breast cancer	CDKN1A	ENSG00000124762	1026
Breast cancer	HEY1	ENSG00000164683	23462
Breast cancer	TP53	ENSG00000141510	7157
Breast cancer	GADD45A	ENSG00000116717	1647
Viral carcinogenesis	TP53	ENSG00000141510	7157
Viral carcinogenesis	PMAIP1	ENSG00000141682	5366
Viral carcinogenesis	PIK3R3	ENSG00000117461	8503
Viral carcinogenesis	EGR2	ENSG00000122877	1959
Viral carcinogenesis	CDKN1A	ENSG00000124762	1026
Viral carcinogenesis	HDAC4	ENSG00000068024	9759
Viral carcinogenesis	IL6ST	ENSG00000134352	3572
Viral carcinogenesis	DDX3X	ENSG00000215301	1654
Viral carcinogenesis	HDAC9	ENSG00000048052	9734
Non-alcoholic fatty liver disease	SOCS3	ENSG00000184557	9021
Non-alcoholic fatty liver disease	PIK3R3	ENSG00000117461	8503
Non-alcoholic fatty liver disease	BCL2L11	ENSG00000153094	10018
Non-alcoholic fatty liver disease	ERN1	ENSG00000178607	2081

Non-alcoholic fatty liver disease	IL6R	ENSG00000160712	3570
Non-alcoholic fatty liver disease	TNFRSF1A	ENSG00000067182	7132
Non-alcoholic fatty liver disease	MAP3K5	ENSG00000197442	4217
Non-alcoholic fatty liver disease	NDUFC1	ENSG00000109390	4717
Glioma	BRAF	ENSG00000157764	673
Glioma	PIK3R3	ENSG00000117461	8503
Glioma	TP53	ENSG00000141510	7157
Glioma	E2F3	ENSG00000112242	1871
Glioma	CDKN1A	ENSG00000124762	1026
Glioma	GADD45A	ENSG00000116717	1647
Melanoma	BRAF	ENSG00000157764	673
Melanoma	CDKN1A	ENSG00000124762	1026
Melanoma	TP53	ENSG00000141510	7157
Melanoma	E2F3	ENSG00000112242	1871
Melanoma	PIK3R3	ENSG00000117461	8503
Melanoma	GADD45A	ENSG00000116717	1647
EGFR tyrosine kinase inhibitor	PIK3R3	ENSG00000117461	8503
EGFR tyrosine kinase inhibitor	BRAF	ENSG00000157764	673
EGFR tyrosine kinase inhibitor	BCL2L11	ENSG00000153094	10018
EGFR tyrosine kinase inhibitor	IL6R	ENSG00000160712	3570
EGFR tyrosine kinase inhibitor	NRG2	ENSG00000158458	9542
EGFR tyrosine kinase inhibitor	FOXO3	ENSG00000118689	2309
Platinum drug resistance	TP53	ENSG00000141510	7157
Platinum drug resistance	PMAIP1	ENSG00000141682	5366
Platinum drug resistance	PIK3R3	ENSG00000117461	8503
Platinum drug resistance	CDKN1A	ENSG00000124762	1026
Platinum drug resistance	GSTM5	ENSG00000134201	2949
Platinum drug resistance	MGST3	ENSG00000143198	4259
Platinum drug resistance	MAP3K5	ENSG00000197442	4217
NF-kappa B signaling pathway	IL1R1	ENSG00000115594	3554
NF-kappa B signaling pathway	TNFRSF1A	ENSG00000067182	7132
NF-kappa B signaling pathway	MAP3K14	ENSG00000006062	9020
NF-kappa B signaling pathway	PLAU	ENSG00000122861	5328
NF-kappa B signaling pathway	CFLAR	ENSG00000003402	8837
NF-kappa B signaling pathway	GADD45A	ENSG00000116717	1647
NF-kappa B signaling pathway	CXCL2	ENSG00000081041	2920
Amoebiasis	CXCL2	ENSG00000081041	2920
Amoebiasis	IL1R1	ENSG00000115594	3554
Amoebiasis	PIK3R3	ENSG00000117461	8503
Amoebiasis	TGFB3	ENSG00000119699	7043
Amoebiasis	ARG2	ENSG00000081181	384
Epstein-Barr virus infection	TP53	ENSG00000141510	7157
Epstein-Barr virus infection	MAP3K14	ENSG00000006062	9020
Epstein-Barr virus infection	PIK3R3	ENSG00000117461	8503
Epstein-Barr virus infection	CDKN1A	ENSG00000124762	1026
Epstein-Barr virus infection	GADD45A	ENSG00000116717	1647
Epstein-Barr virus infection	E2F3	ENSG00000112242	1871
Epstein-Barr virus infection	BCL2L11	ENSG00000153094	10018
Small cell lung cancer	PIK3R3	ENSG00000117461	8503
Small cell lung cancer	E2F3	ENSG00000112242	1871
Small cell lung cancer	TP53	ENSG00000141510	7157
Small cell lung cancer	CDKN1A	ENSG00000124762	1026
Small cell lung cancer	GADD45A	ENSG00000116717	1647
Longevity regulating pathway	PIK3R3	ENSG00000117461	8503
Longevity regulating pathway	TP53	ENSG00000141510	7157
Longevity regulating pathway	FOXO3	ENSG00000118689	2309
Longevity regulating pathway	SESN1	ENSG00000080546	27244
Longevity regulating pathway	APPL1	ENSG00000157500	26060
MicroRNAs in cancer	CYP1B1	ENSG00000138061	1545

MicroRNAs in cancer	HDAC4	ENSG00000068024	9759
MicroRNAs in cancer	HMOX1	ENSG00000100292	3162
MicroRNAs in cancer	PIK3R3	ENSG00000117461	8503
MicroRNAs in cancer	E2F3	ENSG00000112242	1871
MicroRNAs in cancer	NOTCH1	ENSG00000148400	4851
MicroRNAs in cancer	BCL2L11	ENSG00000153094	10018
MicroRNAs in cancer	CDCA5	ENSG00000146670	113130
MicroRNAs in cancer	CDKN1A	ENSG00000124762	1026
MicroRNAs in cancer	SOX4	ENSG00000124766	6659
MicroRNAs in cancer	ITGA5	ENSG00000161638	3678
MicroRNAs in cancer	PLAU	ENSG00000122861	5328
MicroRNAs in cancer	ABCC1	ENSG00000103222	4363
MicroRNAs in cancer	TP53	ENSG00000141510	7157
MicroRNAs in cancer	BMF	ENSG00000104081	90427
MicroRNAs in cancer	TPM1	ENSG00000140416	7168
MicroRNAs in cancer	PRKCE	ENSG00000171132	5581
Alcoholic liver disease	CPT1A	ENSG00000110090	1374
Alcoholic liver disease	ACACB	ENSG00000076555	32
Alcoholic liver disease	MLYCD	ENSG00000103150	23417
Alcoholic liver disease	TNFRSF1A	ENSG00000067182	7132
Alcoholic liver disease	MAP3K14	ENSG00000006062	9020
Alcoholic liver disease	C5AR1	ENSG00000197405	728
Alcoholic liver disease	CXCL2	ENSG00000081041	2920
Alcoholic liver disease	LPIN1	ENSG00000134324	23175
Alcoholic liver disease	ACOX1	ENSG00000161533	51
Alcoholic liver disease	FOXO3	ENSG00000118689	2309
Alcoholic liver disease	MAP3K5	ENSG00000197442	4217
Alcoholic liver disease	ADH1B	ENSG00000196616	125
Hepatocellular carcinoma	BRAF	ENSG00000157764	673
Hepatocellular carcinoma	PIK3R3	ENSG00000117461	8503
Hepatocellular carcinoma	FZD5	ENSG00000163251	7855
Hepatocellular carcinoma	E2F3	ENSG00000112242	1871
Hepatocellular carcinoma	TP53	ENSG00000141510	7157
Hepatocellular carcinoma	CDKN1A	ENSG00000124762	1026
Hepatocellular carcinoma	GADD45A	ENSG00000116717	1647
Hepatocellular carcinoma	ELK1	ENSG00000126767	2002
Hepatocellular carcinoma	TGFB3	ENSG00000119699	7043
Hepatocellular carcinoma	HMOX1	ENSG00000100292	3162
Hepatocellular carcinoma	TXNRD1	ENSG00000198431	7296
Hepatocellular carcinoma	GSTM5	ENSG00000134201	2949
Hepatocellular carcinoma	MGST3	ENSG00000143198	4259
Axon guidance	UNC5B	ENSG00000107731	219699
Axon guidance	EFNB2	ENSG00000125266	1948
Axon guidance	EPHB4	ENSG00000196411	2050
Axon guidance	SEMA7A	ENSG00000138623	8482
Axon guidance	SEMA3G	ENSG00000010319	56920
Axon guidance	SEMA6B	ENSG00000167680	10501
Axon guidance	SEMA4B	ENSG00000185033	10509
Axon guidance	SEMA4C	ENSG00000168758	54910
Axon guidance	PIK3R3	ENSG00000117461	8503
Axon guidance	GNAI1	ENSG00000127955	2770
Axon guidance	PPP3CA	ENSG00000138814	5530
Axon guidance	EPHB1	ENSG00000154928	2047
Axon guidance	SRGAP3	ENSG00000196220	9901
Adipocytokine signaling pathwa	SOCS3	ENSG00000184557	9021
Adipocytokine signaling pathwa	CPT1A	ENSG00000110090	1374
Adipocytokine signaling pathwa	ACSL1	ENSG00000151726	2180
Adipocytokine signaling pathwa	ACSL3	ENSG00000123983	2181
Adipocytokine signaling pathwa	ACACB	ENSG00000076555	32

Adipocytokine signaling pathwa	TNFRSF1B	ENSG00000028137	7133
Adipocytokine signaling pathwa	TNFRSF1A	ENSG00000067182	7132
Adipocytokine signaling pathwa	ACSL6	ENSG00000164398	23305
Adipocytokine signaling pathwa	APPL1	ENSG00000157500	26060
ErbB signaling pathway	ELK1	ENSG00000126767	2002
ErbB signaling pathway	CDKN1A	ENSG00000124762	1026
ErbB signaling pathway	NRG2	ENSG00000158458	9542
ErbB signaling pathway	ABL2	ENSG00000143322	27
ErbB signaling pathway	PIK3R3	ENSG00000117461	8503
ErbB signaling pathway	BRAF	ENSG00000157764	673
Proteoglycans in cancer	PIK3R3	ENSG00000117461	8503
Proteoglycans in cancer	ELK1	ENSG00000126767	2002
Proteoglycans in cancer	BRAF	ENSG00000157764	673
Proteoglycans in cancer	DDX5	ENSG00000108654	1655
Proteoglycans in cancer	TP53	ENSG00000141510	7157
Proteoglycans in cancer	PLAU	ENSG00000122861	5328
Proteoglycans in cancer	PLAUR	ENSG00000011422	5329
Proteoglycans in cancer	CDKN1A	ENSG00000124762	1026
Proteoglycans in cancer	FZD5	ENSG00000163251	7855
Proteoglycans in cancer	ITGA5	ENSG00000161638	3678
Proteoglycans in cancer	CAV1	ENSG00000105974	857
Shigellosis	TNFRSF1A	ENSG00000067182	7132
Shigellosis	IL1R1	ENSG00000115594	3554
Shigellosis	MAP1LC3B2	ENSG00000258102	643246
Shigellosis	ITGA5	ENSG00000161638	3678
Shigellosis	PIK3R3	ENSG00000117461	8503
Shigellosis	TP53	ENSG00000141510	7157
Shigellosis	HK2	ENSG00000159399	3099
Shigellosis	PRKCE	ENSG00000171132	5581
Shigellosis	RPS27A	ENSG00000143947	6233
Shigellosis	FOXO3	ENSG00000118689	2309
Chemical carcinogenesis - reac	PIK3R3	ENSG00000117461	8503
Chemical carcinogenesis - reac	BRAF	ENSG00000157764	673
Chemical carcinogenesis - reac	MAP3K14	ENSG00000006062	9020
Chemical carcinogenesis - reac	ABL2	ENSG00000143322	27
Chemical carcinogenesis - reac	CYP1B1	ENSG00000138061	1545
Chemical carcinogenesis - reac	HMOX1	ENSG00000100292	3162
Chemical carcinogenesis - reac	NDUFC1	ENSG00000109390	4717
Chemical carcinogenesis - reac	FOXO3	ENSG00000118689	2309
Chemical carcinogenesis - reac	MAP3K5	ENSG00000197442	4217
Chemical carcinogenesis - reac	GSTM5	ENSG00000134201	2949
Chemical carcinogenesis - reac	MGST3	ENSG00000143198	4259
Acute myeloid leukemia	RARA	ENSG00000131759	5914
Acute myeloid leukemia	RUNX1	ENSG00000159216	861
Acute myeloid leukemia	PIK3R3	ENSG00000117461	8503
Acute myeloid leukemia	BRAF	ENSG00000157764	673
Acute myeloid leukemia	PER2	ENSG00000132326	8864
Osteoclast differentiation	CSF1	ENSG00000184371	1435
Osteoclast differentiation	MAP3K14	ENSG00000006062	9020
Osteoclast differentiation	PIK3R3	ENSG00000117461	8503
Osteoclast differentiation	IL1R1	ENSG00000115594	3554
Osteoclast differentiation	FOSB	ENSG00000125740	2354
Osteoclast differentiation	FOSL2	ENSG00000075426	2355
Osteoclast differentiation	TNFRSF1A	ENSG00000067182	7132
Osteoclast differentiation	SOCS3	ENSG00000184557	9021
Osteoclast differentiation	PPP3CA	ENSG00000138814	5530
HIF-1 signaling pathway	PIK3R3	ENSG00000117461	8503
HIF-1 signaling pathway	HK2	ENSG00000159399	3099
HIF-1 signaling pathway	PFKFB3	ENSG00000170525	5209

HIF-1 signaling pathway	NOS3	ENSG00000164867	4846
HIF-1 signaling pathway	HMOX1	ENSG00000100292	3162
HIF-1 signaling pathway	IL6R	ENSG00000160712	3570
HIF-1 signaling pathway	CDKN1A	ENSG00000124762	1026
HIF-1 signaling pathway	GAPDH	ENSG00000111640	2597
HIF-1 signaling pathway	ANGPT1	ENSG00000154188	284
Bacterial invasion of epithelial c	ITGA5	ENSG00000161638	3678
Bacterial invasion of epithelial c	PIK3R3	ENSG00000117461	8503
Bacterial invasion of epithelial c	CLTC	ENSG00000141367	1213
Bacterial invasion of epithelial c	CAV1	ENSG00000105974	857
p53 signaling pathway	GADD45A	ENSG00000116717	1647
p53 signaling pathway	CDKN1A	ENSG00000124762	1026
p53 signaling pathway	TP53	ENSG00000141510	7157
p53 signaling pathway	PMAIP1	ENSG00000141682	5366
p53 signaling pathway	SESN1	ENSG00000080546	27244
Homologous recombination	TOP3A	ENSG00000177302	7156
Homologous recombination	RAD52	ENSG00000002016	5893
Bladder cancer	CDKN1A	ENSG00000124762	1026
Bladder cancer	TP53	ENSG00000141510	7157
Bladder cancer	E2F3	ENSG00000112242	1871
Bladder cancer	BRAF	ENSG00000157764	673
Focal adhesion	ITGA5	ENSG00000161638	3678
Focal adhesion	VASP	ENSG00000125753	7408
Focal adhesion	PIK3R3	ENSG00000117461	8503
Focal adhesion	PIP5K1A	ENSG00000143398	8394
Focal adhesion	ELK1	ENSG00000126767	2002
Focal adhesion	BRAF	ENSG00000157764	673
Focal adhesion	ITGB6	ENSG00000115221	3694
Focal adhesion	CAV1	ENSG00000105974	857
Coronavirus disease - COVID-1	IL6R	ENSG00000160712	3570
Coronavirus disease - COVID-1	MX1	ENSG00000157601	4599
Coronavirus disease - COVID-1	MX2	ENSG00000183486	4600
Coronavirus disease - COVID-1	ADAR	ENSG00000160710	103
Coronavirus disease - COVID-1	CCL2	ENSG00000108691	6347
Coronavirus disease - COVID-1	TNFRSF1A	ENSG00000067182	7132
Coronavirus disease - COVID-1	IL6ST	ENSG00000134352	3572
Coronavirus disease - COVID-1	C5AR1	ENSG00000197405	728
Coronavirus disease - COVID-1	PIK3R3	ENSG00000117461	8503
Coronavirus disease - COVID-1	RPS3A	ENSG00000145425	6189
Coronavirus disease - COVID-1	RPS15	ENSG00000115268	6209
Coronavirus disease - COVID-1	RPS27A	ENSG00000143947	6233
Coronavirus disease - COVID-1	RPS28	ENSG00000233927	6234
Coronavirus disease - COVID-1	RPL10	ENSG00000147403	6134
Coronavirus disease - COVID-1	RPL27A	ENSG00000166441	6157
Huntington disease	HTT	ENSG00000197386	3064
Huntington disease	POLR2A	ENSG00000181222	5430
Huntington disease	TP53	ENSG00000141510	7157
Huntington disease	CLTC	ENSG00000141367	1213
Huntington disease	ERN1	ENSG00000178607	2081
Huntington disease	NDUFC1	ENSG00000109390	4717
Huntington disease	MAP3K5	ENSG00000197442	4217
Huntington disease	TUBA4A	ENSG00000127824	7277
Signaling pathways regulating p	FZD5	ENSG00000163251	7855
Signaling pathways regulating p	IL6ST	ENSG00000134352	3572
Signaling pathways regulating p	KLF4	ENSG00000136826	9314
Signaling pathways regulating p	PIK3R3	ENSG00000117461	8503
Signaling pathways regulating p	TBX3	ENSG00000135111	6926
Signaling pathways regulating p	ID2	ENSG00000115738	3398
Signaling pathways regulating p	ESRRB	ENSG00000119715	2103

Signaling pathways regulating p	ZFHX3	ENSG00000140836	463
Signaling pathways regulating p	LIFR	ENSG00000113594	3977
Signaling pathways regulating p	MEIS1	ENSG00000143995	4211
Gastric cancer	BRAF	ENSG00000157764	673
Gastric cancer	PIK3R3	ENSG00000117461	8503
Gastric cancer	FZD5	ENSG00000163251	7855
Gastric cancer	TP53	ENSG00000141510	7157
Gastric cancer	CDKN1A	ENSG00000124762	1026
Gastric cancer	GADD45A	ENSG00000116717	1647
Gastric cancer	TGFB3	ENSG00000119699	7043
Gastric cancer	E2F3	ENSG00000112242	1871
Ribosome	RPS15	ENSG00000115268	6209
Ribosome	RPS27A	ENSG00000143947	6233
Ribosome	RPS28	ENSG00000233927	6234
Ribosome	RPS3A	ENSG00000145425	6189
Ribosome	RPL27A	ENSG00000166441	6157
Ribosome	RPL10	ENSG00000147403	6134
Ribosome	MRPS6	ENSG00000243927	64968
Parathyroid hormone synthesis	PDE4B	ENSG00000184588	5142
Parathyroid hormone synthesis	PDE4D	ENSG00000113448	5144
Parathyroid hormone synthesis	BRAF	ENSG00000157764	673
Parathyroid hormone synthesis	CDKN1A	ENSG00000124762	1026
Parathyroid hormone synthesis	NR4A2	ENSG00000153234	4929
Parathyroid hormone synthesis	SGK1	ENSG00000118515	6446
Parathyroid hormone synthesis	GNAI1	ENSG00000127955	2770
VEGF signaling pathway	SPHK2	ENSG00000063176	56848
VEGF signaling pathway	PIK3R3	ENSG00000117461	8503
VEGF signaling pathway	NOS3	ENSG00000164867	4846
VEGF signaling pathway	PPP3CA	ENSG00000138814	5530
cGMP-PKG signaling pathway	VASP	ENSG00000125753	7408
cGMP-PKG signaling pathway	NOS3	ENSG00000164867	4846
cGMP-PKG signaling pathway	PPP3CA	ENSG00000138814	5530
cGMP-PKG signaling pathway	PRKCE	ENSG00000171132	5581
cGMP-PKG signaling pathway	GNAI1	ENSG00000127955	2770
Basal cell carcinoma	FZD5	ENSG00000163251	7855
Basal cell carcinoma	TP53	ENSG00000141510	7157
Basal cell carcinoma	CDKN1A	ENSG00000124762	1026
Basal cell carcinoma	GADD45A	ENSG00000116717	1647
Renal cell carcinoma	TGFB3	ENSG00000119699	7043
Renal cell carcinoma	PIK3R3	ENSG00000117461	8503
Renal cell carcinoma	BRAF	ENSG00000157764	673
Renal cell carcinoma	CDKN1A	ENSG00000124762	1026
Viral life cycle - HIV-1	MX1	ENSG00000157601	4599
Viral life cycle - HIV-1	MX2	ENSG00000183486	4600
Viral life cycle - HIV-1	AFF4	ENSG00000072364	27125
Viral life cycle - HIV-1	VPS4B	ENSG00000119541	9525
Viral life cycle - HIV-1	NELFCD	ENSG00000101158	51497
Viral life cycle - HIV-1	MLLT3	ENSG00000171843	4300
Autophagy - animal	PIK3R3	ENSG00000117461	8503
Autophagy - animal	ERN1	ENSG00000178607	2081
Autophagy - animal	CFLAR	ENSG00000003402	8837
Autophagy - animal	GABARAPL1	ENSG00000139112	23710
Autophagy - animal	MAP1LC3B2	ENSG00000258102	643246
Autophagy - animal	WDFY3	ENSG00000163625	23001
Autophagy - animal	PLEKHM1	ENSG00000225190	9842
Autophagy - animal	MTMR4	ENSG00000108389	9110
Autophagy - animal	RPS27A	ENSG00000143947	6233
Fatty acid biosynthesis	ACACB	ENSG00000076555	32
Fatty acid biosynthesis	ACSL1	ENSG00000151726	2180

Fatty acid biosynthesis	ACSL3	ENSG00000123983	2181
Fatty acid biosynthesis	ACSL6	ENSG00000164398	23305
Viral protein interaction with cyt	CCR1	ENSG00000163823	1230
Viral protein interaction with cyt	CCL2	ENSG00000108691	6347
Viral protein interaction with cyt	CXCL2	ENSG00000081041	2920
Viral protein interaction with cyt	ACKR3	ENSG00000144476	57007
Viral protein interaction with cyt	IL6ST	ENSG00000134352	3572
Viral protein interaction with cyt	IL6R	ENSG00000160712	3570
Viral protein interaction with cyt	CSF1	ENSG00000184371	1435
Viral protein interaction with cyt	TNFRSF1A	ENSG00000067182	7132
Viral protein interaction with cyt	TNFRSF1B	ENSG00000028137	7133
Viral protein interaction with cyt	TNFRSF10D	ENSG00000173530	8793
Inflammatory mediator regulatic	IL1R1	ENSG00000115594	3554
Inflammatory mediator regulatic	PIK3R3	ENSG00000117461	8503
Inflammatory mediator regulatic	PRKCE	ENSG00000171132	5581
Diabetic cardiomyopathy	TGFB3	ENSG00000119699	7043
Diabetic cardiomyopathy	PIK3R3	ENSG00000117461	8503
Diabetic cardiomyopathy	NOS3	ENSG00000164867	4846
Diabetic cardiomyopathy	NDUFC1	ENSG00000109390	4717
Diabetic cardiomyopathy	GAPDH	ENSG00000111640	2597
Cytokine-cytokine receptor inte	CSF2RB	ENSG00000100368	1439
Cytokine-cytokine receptor inte	IL6ST	ENSG00000134352	3572
Cytokine-cytokine receptor inte	IL1R1	ENSG00000115594	3554
Cytokine-cytokine receptor inte	TGFB3	ENSG00000119699	7043
Cytokine-cytokine receptor inte	THPO	ENSG00000090534	7066
Cytokine-cytokine receptor inte	OSMR	ENSG00000145623	9180
Cytokine-cytokine receptor inte	IL6R	ENSG00000160712	3570
Cytokine-cytokine receptor inte	TNFRSF1A	ENSG00000067182	7132
Cytokine-cytokine receptor inte	TNFRSF1B	ENSG00000028137	7133
Cytokine-cytokine receptor inte	TNFRSF10D	ENSG00000173530	8793
Cytokine-cytokine receptor inte	CXCL2	ENSG00000081041	2920
Cytokine-cytokine receptor inte	ACKR3	ENSG00000144476	57007
Cytokine-cytokine receptor inte	CCR1	ENSG00000163823	1230
Cytokine-cytokine receptor inte	CCL2	ENSG00000108691	6347
Cytokine-cytokine receptor inte	CSF1	ENSG00000184371	1435
Cytokine-cytokine receptor inte	LIFR	ENSG00000113594	3977
Chagas disease	TNFRSF1A	ENSG00000067182	7132
Chagas disease	PIK3R3	ENSG00000117461	8503
Chagas disease	TGFB3	ENSG00000119699	7043
Chagas disease	CFLAR	ENSG00000003402	8837
Chagas disease	CCL2	ENSG00000108691	6347
Chagas disease	GNAI1	ENSG00000127955	2770
Insulin resistance	TNFRSF1A	ENSG00000067182	7132
Insulin resistance	PIK3R3	ENSG00000117461	8503
Insulin resistance	SOCS3	ENSG00000184557	9021
Insulin resistance	PPP1R3B	ENSG00000173281	79660
Insulin resistance	NOS3	ENSG00000164867	4846
Insulin resistance	ACACB	ENSG00000076555	32
Insulin resistance	CPT1A	ENSG00000110090	1374
Insulin resistance	PRKCE	ENSG00000171132	5581
Prolactin signaling pathway	PIK3R3	ENSG00000117461	8503
Prolactin signaling pathway	SOCS3	ENSG00000184557	9021
Prolactin signaling pathway	FOXO3	ENSG00000118689	2309
Th17 cell differentiation	IL1R1	ENSG00000115594	3554
Th17 cell differentiation	IL6R	ENSG00000160712	3570
Th17 cell differentiation	IL6ST	ENSG00000134352	3572
Th17 cell differentiation	RUNX1	ENSG00000159216	861
Th17 cell differentiation	RARA	ENSG00000131759	5914
Th17 cell differentiation	PPP3CA	ENSG00000138814	5530

Pathogenic Escherichia coli infe	CLDN5	ENSG00000184113	7122
Pathogenic Escherichia coli infe	TNFRSF1A	ENSG00000067182	7132
Pathogenic Escherichia coli infe	IL1R1	ENSG00000115594	3554
Pathogenic Escherichia coli infe	MYO1E	ENSG00000157483	4643
Pathogenic Escherichia coli infe	TUBA4A	ENSG00000127824	7277
Pathogenic Escherichia coli infe	GAPDH	ENSG00000111640	2597
Cell cycle	CDKN1A	ENSG00000124762	1026
Cell cycle	GADD45A	ENSG00000116717	1647
Cell cycle	TP53	ENSG00000141510	7157
Cell cycle	TGFB3	ENSG00000119699	7043
Cell cycle	E2F3	ENSG00000112242	1871
Cell cycle	CDCA5	ENSG00000146670	113130
Cell cycle	CDC26	ENSG00000176386	246184
Amphetamine addiction	FOSB	ENSG00000125740	2354
Amphetamine addiction	PPP3CA	ENSG00000138814	5530
Cushing syndrome	LDLR	ENSG00000130164	3949
Cushing syndrome	NCEH1	ENSG00000144959	57552
Cushing syndrome	FZD5	ENSG00000163251	7855
Cushing syndrome	CDKN1A	ENSG00000124762	1026
Cushing syndrome	E2F3	ENSG00000112242	1871
Cushing syndrome	RBBP5	ENSG00000117222	5929
Cushing syndrome	BRAF	ENSG00000157764	673
Cushing syndrome	GNAI1	ENSG00000127955	2770
GABAergic synapse	GABARAPL1	ENSG00000139112	23710
GABAergic synapse	GNAI1	ENSG00000127955	2770
GABAergic synapse	GNG11	ENSG00000127920	2791
GABAergic synapse	SLC38A3	ENSG00000188338	10991
Estrogen signaling pathway	PIK3R3	ENSG00000117461	8503
Estrogen signaling pathway	NOS3	ENSG00000164867	4846
Estrogen signaling pathway	NCOA1	ENSG00000084676	8648
Estrogen signaling pathway	RARA	ENSG00000131759	5914
Estrogen signaling pathway	HSPA1L	ENSG00000204390	3305
Estrogen signaling pathway	GNAI1	ENSG00000127955	2770
Spinocerebellar ataxia	ERN1	ENSG00000178607	2081
Spinocerebellar ataxia	PIK3R3	ENSG00000117461	8503
Spinocerebellar ataxia	MAP3K5	ENSG00000197442	4217
Protein processing in endoplasi	MAN1A2	ENSG00000198162	10905
Protein processing in endoplasi	ATF6	ENSG00000118217	22926
Protein processing in endoplasi	ERN1	ENSG00000178607	2081
Protein processing in endoplasi	YOD1	ENSG00000180667	55432
Protein processing in endoplasi	MAP3K5	ENSG00000197442	4217
Protein processing in endoplasi	HSPA1L	ENSG00000204390	3305
Necroptosis	TNFRSF1A	ENSG00000067182	7132
Necroptosis	CFLAR	ENSG00000003402	8837
Necroptosis	VPS4B	ENSG00000119541	9525
Central carbon metabolism in c	PIK3R3	ENSG00000117461	8503
Central carbon metabolism in c	HK2	ENSG00000159399	3099
Central carbon metabolism in c	TP53	ENSG00000141510	7157
Central carbon metabolism in c	SLC7A5	ENSG00000103257	8140
mTOR signaling pathway	PIK3R3	ENSG00000117461	8503
mTOR signaling pathway	BRAF	ENSG00000157764	673
mTOR signaling pathway	LPIN1	ENSG00000134324	23175
mTOR signaling pathway	SGK1	ENSG00000118515	6446
mTOR signaling pathway	TNFRSF1A	ENSG00000067182	7132
mTOR signaling pathway	SLC7A5	ENSG00000103257	8140
mTOR signaling pathway	FZD5	ENSG00000163251	7855
mTOR signaling pathway	CLIP1	ENSG00000130779	6249
Complement and coagulation c	PLAU	ENSG00000122861	5328
Complement and coagulation c	PLAUR	ENSG00000011422	5329

Complement and coagulation c	C5AR1	ENSG00000197405	728
Complement and coagulation c	CD55	ENSG00000196352	1604
Complement and coagulation c	SERPINF2	ENSG00000167711	5345
Complement and coagulation c	PLAT	ENSG00000104368	5327
Mitophagy - animal	TP53	ENSG00000141510	7157
Mitophagy - animal	GABARAPL1	ENSG00000139112	23710
Mitophagy - animal	MAP1LC3B2	ENSG00000258102	643246
Mitophagy - animal	RPS27A	ENSG00000143947	6233
Mitophagy - animal	FOXO3	ENSG00000118689	2309
C-type lectin receptor signaling	BCL3	ENSG00000069399	602
C-type lectin receptor signaling	MAP3K14	ENSG00000006062	9020
C-type lectin receptor signaling	PIK3R3	ENSG00000117461	8503
C-type lectin receptor signaling	EGR2	ENSG00000122877	1959
C-type lectin receptor signaling	PPP3CA	ENSG00000138814	5530
Parkinson disease	ERN1	ENSG00000178607	2081
Parkinson disease	ATF6	ENSG00000118217	22926
Parkinson disease	TP53	ENSG00000141510	7157
Parkinson disease	RPS27A	ENSG00000143947	6233
Parkinson disease	NDUFC1	ENSG00000109390	4717
Parkinson disease	GNAI1	ENSG00000127955	2770
Parkinson disease	TUBA4A	ENSG00000127824	7277
Parkinson disease	MAP3K5	ENSG00000197442	4217
Spliceosome	DDX5	ENSG00000108654	1655
Spliceosome	SF3A3	ENSG00000183431	10946
Spliceosome	SRSF3	ENSG00000112081	6428
Spliceosome	AQR	ENSG00000021776	9716
Spliceosome	HSPA1L	ENSG00000204390	3305
Spliceosome	LSM4	ENSG00000130520	25804
Aldosterone-regulated sodium r	SGK1	ENSG00000118515	6446
Aldosterone-regulated sodium r	PIK3R3	ENSG00000117461	8503
Aldosterone-regulated sodium r	NR3C2	ENSG00000151623	4306
Influenza A	PIK3R3	ENSG00000117461	8503
Influenza A	MX1	ENSG00000157601	4599
Influenza A	MX2	ENSG00000183486	4600
Influenza A	ADAR	ENSG00000160710	103
Influenza A	SOCS3	ENSG00000184557	9021
Influenza A	KPNA2	ENSG00000182481	3838
Influenza A	CCL2	ENSG00000108691	6347
Influenza A	TNFRSF1A	ENSG00000067182	7132
Influenza A	KPNA5	ENSG00000196911	3841
Growth hormone synthesis, sec	PIK3R3	ENSG00000117461	8503
Growth hormone synthesis, sec	SOCS3	ENSG00000184557	9021
Growth hormone synthesis, sec	GNAI1	ENSG00000127955	2770
Lysosome	CLTC	ENSG00000141367	1213
Lysosome	AP1G1	ENSG00000166747	164
Lysosome	AP4E1	ENSG00000081014	23431
Thyroid hormone signaling path	PIK3R3	ENSG00000117461	8503
Thyroid hormone signaling path	TP53	ENSG00000141510	7157
Thyroid hormone signaling path	RCAN2	ENSG00000172348	10231
Thyroid hormone signaling path	NCOA1	ENSG00000084676	8648
Thyroid hormone signaling path	NOTCH1	ENSG00000148400	4851
Measles	PIK3R3	ENSG00000117461	8503
Measles	MX1	ENSG00000157601	4599
Measles	MX2	ENSG00000183486	4600
Measles	TP53	ENSG00000141510	7157
Measles	ADAR	ENSG00000160710	103
Measles	HSPA1L	ENSG00000204390	3305
Chemokine signaling pathway	CCR1	ENSG00000163823	1230
Chemokine signaling pathway	CXCL2	ENSG00000081041	2920

Chemokine signaling pathway	CCL2	ENSG00000108691	6347
Chemokine signaling pathway	PIK3R3	ENSG00000117461	8503
Chemokine signaling pathway	BRAF	ENSG00000157764	673
Chemokine signaling pathway	GNAI1	ENSG00000127955	2770
Chemokine signaling pathway	GNG11	ENSG00000127920	2791
Chemokine signaling pathway	FOXO3	ENSG00000118689	2309
Human immunodeficiency virus	PIK3R3	ENSG00000117461	8503
Human immunodeficiency virus	TNFRSF1A	ENSG00000067182	7132
Human immunodeficiency virus	AP1G1	ENSG00000166747	164
Human immunodeficiency virus	TNFRSF1B	ENSG00000028137	7133
Human immunodeficiency virus	GNG11	ENSG00000127920	2791
Human immunodeficiency virus	PPP3CA	ENSG00000138814	5530
Human immunodeficiency virus	GNAI1	ENSG00000127955	2770
Chemical carcinogenesis - rece	PIK3R3	ENSG00000117461	8503
Chemical carcinogenesis - rece	CYP1B1	ENSG00000138061	1545
Chemical carcinogenesis - rece	KPNA2	ENSG00000182481	3838
Chemical carcinogenesis - rece	KLF4	ENSG00000136826	9314
Chemical carcinogenesis - rece	DLL1	ENSG00000198719	28514
Chemical carcinogenesis - rece	GSTM5	ENSG00000134201	2949
Chemical carcinogenesis - rece	MGST3	ENSG00000143198	4259
Chemical carcinogenesis - rece	KPNA5	ENSG00000196911	3841
Chemical carcinogenesis - rece	GNAI1	ENSG00000127955	2770
Wnt signaling pathway	FZD5	ENSG00000163251	7855
Wnt signaling pathway	PRICKLE2	ENSG00000163637	166336
Wnt signaling pathway	SOX17	ENSG00000164736	64321
Wnt signaling pathway	CHD8	ENSG00000100888	57680
Wnt signaling pathway	TP53	ENSG00000141510	7157
Wnt signaling pathway	PSEN1	ENSG00000080815	5663
Wnt signaling pathway	PPP3CA	ENSG00000138814	5530
Wnt signaling pathway	DAAM1	ENSG00000100592	23002
Wnt signaling pathway	GPC4	ENSG00000076716	2239
Wnt signaling pathway	TLE1	ENSG00000196781	7088
Wnt signaling pathway	MCC	ENSG00000171444	4163
Type II diabetes mellitus	PIK3R3	ENSG00000117461	8503
Type II diabetes mellitus	SOCS3	ENSG00000184557	9021
Type II diabetes mellitus	HK2	ENSG00000159399	3099
Type II diabetes mellitus	PRKCE	ENSG00000171132	5581
Notch signaling pathway	PSEN1	ENSG00000080815	5663
Notch signaling pathway	NOTCH1	ENSG00000148400	4851
Notch signaling pathway	DLL1	ENSG00000198719	28514
Notch signaling pathway	HEY1	ENSG00000164683	23462
Notch signaling pathway	TLE1	ENSG00000196781	7088
Th1 and Th2 cell differentiation	NOTCH1	ENSG00000148400	4851
Th1 and Th2 cell differentiation	DLL1	ENSG00000198719	28514
Th1 and Th2 cell differentiation	PPP3CA	ENSG00000138814	5530
Efferocytosis	ABCA1	ENSG00000165029	19
Efferocytosis	CPT1A	ENSG00000110090	1374
Efferocytosis	DUSP2	ENSG00000158050	1844
Efferocytosis	SGK1	ENSG00000118515	6446
Efferocytosis	SPHK2	ENSG00000063176	56848
Efferocytosis	RAB17	ENSG00000124839	64284
Efferocytosis	VPS8	ENSG00000156931	23355
Efferocytosis	ARG2	ENSG00000081181	384
Efferocytosis	DUSP7	ENSG00000164086	1849
Toxoplasmosis	TNFRSF1A	ENSG00000067182	7132
Toxoplasmosis	LDLR	ENSG00000130164	3949
Toxoplasmosis	TGFB3	ENSG00000119699	7043
Toxoplasmosis	GNAI1	ENSG00000127955	2770
Toxoplasmosis	HSPA1L	ENSG00000204390	3305

GnRH secretion	PIK3R3	ENSG00000117461	8503
GnRH secretion	HCN1	ENSG00000164588	348980
Carbohydrate digestion and abs	HK2	ENSG00000159399	3099
Carbohydrate digestion and abs	PIK3R3	ENSG00000117461	8503
Platelet activation	PIK3R3	ENSG00000117461	8503
Platelet activation	NOS3	ENSG00000164867	4846
Platelet activation	VASP	ENSG00000125753	7408
Platelet activation	GNAI1	ENSG00000127955	2770
cAMP signaling pathway	PIK3R3	ENSG00000117461	8503
cAMP signaling pathway	BRAF	ENSG00000157764	673
cAMP signaling pathway	PDE4B	ENSG00000184588	5142
cAMP signaling pathway	PDE4D	ENSG00000113448	5144
cAMP signaling pathway	ACOX1	ENSG00000161533	51
cAMP signaling pathway	POPDC2	ENSG00000121577	64091
cAMP signaling pathway	GNAI1	ENSG00000127955	2770
cAMP signaling pathway	RAPGEF3	ENSG00000079337	10411
Apoptosis - multiple species	TNFRSF1A	ENSG00000067182	7132
Apoptosis - multiple species	BCL2L11	ENSG00000153094	10018
Apoptosis - multiple species	PMAIP1	ENSG00000141682	5366
Oxytocin signaling pathway	NOS3	ENSG00000164867	4846
Oxytocin signaling pathway	ELK1	ENSG00000126767	2002
Oxytocin signaling pathway	CDKN1A	ENSG00000124762	1026
Oxytocin signaling pathway	CACNG1	ENSG00000108878	786
Oxytocin signaling pathway	PPP3CA	ENSG00000138814	5530
Oxytocin signaling pathway	GNAI1	ENSG00000127955	2770
Insulin signaling pathway	SOCS3	ENSG00000184557	9021
Insulin signaling pathway	ELK1	ENSG00000126767	2002
Insulin signaling pathway	PPP1R3B	ENSG00000173281	79660
Insulin signaling pathway	ACACB	ENSG00000076555	32
Insulin signaling pathway	BRAF	ENSG00000157764	673
Insulin signaling pathway	PIK3R3	ENSG00000117461	8503
Insulin signaling pathway	HK2	ENSG00000159399	3099
Regulation of lipolysis in adipoc	PIK3R3	ENSG00000117461	8503
Regulation of lipolysis in adipoc	GNAI1	ENSG00000127955	2770
Regulation of lipolysis in adipoc	FABP4	ENSG00000170323	2167
Prion disease	NOTCH1	ENSG00000148400	4851
Prion disease	PIK3R3	ENSG00000117461	8503
Prion disease	HSPA1L	ENSG00000204390	3305
Prion disease	NDUFC1	ENSG00000109390	4717
Prion disease	TUBA4A	ENSG00000127824	7277
Prion disease	PPP3CA	ENSG00000138814	5530
Prion disease	CAV1	ENSG00000105974	857
Longevity regulating pathway -	PIK3R3	ENSG00000117461	8503
Longevity regulating pathway -	EIF4EBP2	ENSG00000148730	1979
Longevity regulating pathway -	FOXO3	ENSG00000118689	2309
Longevity regulating pathway -	HSPA1L	ENSG00000204390	3305
Cytosolic DNA-sensing pathwa	ADAR	ENSG00000160710	103
Cytosolic DNA-sensing pathwa	POLR3A	ENSG00000148606	11128
Cytosolic DNA-sensing pathwa	IFI16	ENSG00000163565	3428
Tight junction	VASP	ENSG00000125753	7408
Tight junction	CLDN5	ENSG00000184113	7122
Tight junction	RUNX1	ENSG00000159216	861
Tight junction	MAP3K5	ENSG00000197442	4217
Tight junction	TUBA4A	ENSG00000127824	7277
Tight junction	PRKCE	ENSG00000171132	5581
Salmonella infection	TNFRSF1A	ENSG00000067182	7132
Salmonella infection	PLEKHM1	ENSG00000225190	9842
Salmonella infection	GAPDH	ENSG00000111640	2597
Salmonella infection	TUBA4A	ENSG00000127824	7277

Salmonella infection	STX10	ENSG00000104915	8677
Ubiquitin mediated proteolysis	SOCS3	ENSG00000184557	9021
Ubiquitin mediated proteolysis	UBR5	ENSG00000104517	51366
Ubiquitin mediated proteolysis	CDC26	ENSG00000176386	246184
Ubiquitin mediated proteolysis	RPS27A	ENSG00000143947	6233
Alcoholism	HDAC4	ENSG00000068024	9759
Alcoholism	BRAF	ENSG00000157764	673
Alcoholism	FOSB	ENSG00000125740	2354
Alcoholism	HDAC9	ENSG00000048052	9734
Alcoholism	GNAI1	ENSG00000127955	2770
Alcoholism	GNG11	ENSG00000127920	2791
Fc epsilon RI signaling pathway	PIK3R3	ENSG00000117461	8503
Fatty acid metabolism	ACOX1	ENSG00000161533	51
Fatty acid metabolism	ACSL1	ENSG00000151726	2180
Fatty acid metabolism	ACSL3	ENSG00000123983	2181
Fatty acid metabolism	CPT1A	ENSG00000110090	1374
Fatty acid metabolism	ACSL6	ENSG00000164398	23305
Endocytosis	STAM	ENSG00000136738	8027
Endocytosis	LDLR	ENSG00000130164	3949
Endocytosis	CLTC	ENSG00000141367	1213
Endocytosis	VPS4B	ENSG00000119541	9525
Endocytosis	PIP5K1A	ENSG00000143398	8394
Endocytosis	VPS28	ENSG00000160948	51160
Endocytosis	HSPA1L	ENSG00000204390	3305
Endocytosis	CAV1	ENSG00000105974	857
NOD-like receptor signaling pat	GBP2	ENSG00000162645	2634
NOD-like receptor signaling pat	GABARAPL1	ENSG00000139112	23710
NOD-like receptor signaling pat	MAP1LC3B2	ENSG00000258102	643246
NOD-like receptor signaling pat	IFI16	ENSG00000163565	3428
NOD-like receptor signaling pat	DHX33	ENSG00000005100	56919
NOD-like receptor signaling pat	CXCL2	ENSG00000081041	2920
NOD-like receptor signaling pat	CCL2	ENSG00000108691	6347
Neutrophil extracellular trap for	PIK3R3	ENSG00000117461	8503
Neutrophil extracellular trap for	HDAC4	ENSG00000068024	9759
Neutrophil extracellular trap for	C5AR1	ENSG00000197405	728
Neutrophil extracellular trap for	HDAC9	ENSG00000048052	9734
B cell receptor signaling pathwa	PIK3R3	ENSG00000117461	8503
B cell receptor signaling pathwa	PPP3CA	ENSG00000138814	5530
B cell receptor signaling pathwa	CD79B	ENSG00000007312	974
Vascular smooth muscle contra	BRAF	ENSG00000157764	673
Vascular smooth muscle contra	PRKCE	ENSG00000171132	5581
PD-L1 expression and PD-1 ch	PIK3R3	ENSG00000117461	8503
PD-L1 expression and PD-1 ch	PPP3CA	ENSG00000138814	5530
Tuberculosis	SPHK2	ENSG00000063176	56848
Tuberculosis	TNFRSF1A	ENSG00000067182	7132
Tuberculosis	TGFB3	ENSG00000119699	7043
Tuberculosis	PPP3CA	ENSG00000138814	5530
Cholinergic synapse	PIK3R3	ENSG00000117461	8503
Cholinergic synapse	ACHE	ENSG00000087085	43
Cholinergic synapse	GNAI1	ENSG00000127955	2770
Cholinergic synapse	GNG11	ENSG00000127920	2791
Choline metabolism in cancer	PIK3R3	ENSG00000117461	8503
Choline metabolism in cancer	PIP5K1A	ENSG00000143398	8394
Rap1 signaling pathway	CSF1	ENSG00000184371	1435
Rap1 signaling pathway	RASSF5	ENSG00000266094	83593
Rap1 signaling pathway	VASP	ENSG00000125753	7408
Rap1 signaling pathway	BRAF	ENSG00000157764	673
Rap1 signaling pathway	RAPGEF5	ENSG00000136237	9771
Rap1 signaling pathway	PIK3R3	ENSG00000117461	8503

Rap1 signaling pathway	ANGPT1	ENSG00000154188	284
Rap1 signaling pathway	RAPGEF3	ENSG00000079337	10411
Rap1 signaling pathway	GNAI1	ENSG00000127955	2770
Fc gamma R-mediated phagoc	PIK3R3	ENSG00000117461	8503
Fc gamma R-mediated phagoc	VASP	ENSG00000125753	7408
Fc gamma R-mediated phagoc	PIP5K1A	ENSG00000143398	8394
Fc gamma R-mediated phagoc	SPHK2	ENSG00000063176	56848
Fc gamma R-mediated phagoc	PRKCE	ENSG00000171132	5581
Apelin signaling pathway	SPHK2	ENSG00000063176	56848
Apelin signaling pathway	KLF2	ENSG00000127528	10365
Apelin signaling pathway	NOS3	ENSG00000164867	4846
Apelin signaling pathway	HDAC4	ENSG00000068024	9759
Apelin signaling pathway	MAP1LC3B2	ENSG00000258102	643246
Apelin signaling pathway	GNAI1	ENSG00000127955	2770
Apelin signaling pathway	GNG11	ENSG00000127920	2791
Apelin signaling pathway	PRKCE	ENSG00000171132	5581
Apelin signaling pathway	PLAT	ENSG00000104368	5327
Phosphatidylinositol signaling s	PIK3R3	ENSG00000117461	8503
Phosphatidylinositol signaling s	PIP5K1A	ENSG00000143398	8394
Phosphatidylinositol signaling s	SYNJ2	ENSG00000078269	8871
Phosphatidylinositol signaling s	IP6K3	ENSG00000161896	117283
Phosphatidylinositol signaling s	MTMR4	ENSG00000108389	9110
Progesterone-mediated oocyte	PIK3R3	ENSG00000117461	8503
Progesterone-mediated oocyte	BRAF	ENSG00000157764	673
Progesterone-mediated oocyte	CDC26	ENSG00000176386	246184
Progesterone-mediated oocyte	GNAI1	ENSG00000127955	2770
AGE-RAGE signaling pathway	TGFB3	ENSG00000119699	7043
AGE-RAGE signaling pathway	PIK3R3	ENSG00000117461	8503
AGE-RAGE signaling pathway	NOS3	ENSG00000164867	4846
AGE-RAGE signaling pathway	CCL2	ENSG00000108691	6347
AGE-RAGE signaling pathway	PRKCE	ENSG00000171132	5581
Fanconi anemia pathway	TOP3A	ENSG00000177302	7156
Toll-like receptor signaling path	PIK3R3	ENSG00000117461	8503
Leukocyte transendothelial mig	CLDN5	ENSG00000184113	7122
Leukocyte transendothelial mig	PIK3R3	ENSG00000117461	8503
Leukocyte transendothelial mig	RASSF5	ENSG00000266094	83593
Leukocyte transendothelial mig	VASP	ENSG00000125753	7408
Leukocyte transendothelial mig	RAPGEF3	ENSG00000079337	10411
Leukocyte transendothelial mig	GNAI1	ENSG00000127955	2770
Regulation of actin cytoskeletor	PIP5K1A	ENSG00000143398	8394
Regulation of actin cytoskeletor	BRAF	ENSG00000157764	673
Regulation of actin cytoskeletor	PIK3R3	ENSG00000117461	8503
Regulation of actin cytoskeletor	ITGA5	ENSG00000161638	3678
Regulation of actin cytoskeletor	ITGB6	ENSG00000115221	3694
Ras signaling pathway	PIK3R3	ENSG00000117461	8503
Ras signaling pathway	CSF1	ENSG00000184371	1435
Ras signaling pathway	ABL2	ENSG00000143322	27
Ras signaling pathway	ELK1	ENSG00000126767	2002
Ras signaling pathway	ETS2	ENSG00000157557	2114
Ras signaling pathway	RASSF5	ENSG00000266094	83593
Ras signaling pathway	RAPGEF5	ENSG00000136237	9771
Ras signaling pathway	ANGPT1	ENSG00000154188	284
Ras signaling pathway	GNG11	ENSG00000127920	2791
Cocaine addiction	FOSB	ENSG00000125740	2354
Cocaine addiction	GNAI1	ENSG00000127955	2770
Epithelial cell signaling in Helico	CXCL2	ENSG00000081041	2920
Epithelial cell signaling in Helico	MAP3K14	ENSG00000006062	9020
Epithelial cell signaling in Helico	CSK	ENSG00000103653	1445
Natural killer cell mediated cyto	PIK3R3	ENSG00000117461	8503

Natural killer cell mediated cyto	BRAF	ENSG00000157764	673
Natural killer cell mediated cyto	PPP3CA	ENSG00000138814	5530
AMPK signaling pathway	PIK3R3	ENSG00000117461	8503
AMPK signaling pathway	CPT1A	ENSG00000110090	1374
AMPK signaling pathway	PFKFB3	ENSG00000170525	5209
AMPK signaling pathway	ACACB	ENSG00000076555	32
AMPK signaling pathway	MLYCD	ENSG00000103150	23417
AMPK signaling pathway	FOXO3	ENSG00000118689	2309
T cell receptor signaling pathwa	MAP3K14	ENSG00000006062	9020
T cell receptor signaling pathwa	PIK3R3	ENSG00000117461	8503
T cell receptor signaling pathwa	PPP3CA	ENSG00000138814	5530
Relaxin signaling pathway	NOS3	ENSG00000164867	4846
Relaxin signaling pathway	PIK3R3	ENSG00000117461	8503
Relaxin signaling pathway	GNG11	ENSG00000127920	2791
Relaxin signaling pathway	GNAI1	ENSG00000127955	2770
Hypertrophic cardiomyopathy	ITGA5	ENSG00000161638	3678
Hypertrophic cardiomyopathy	TGFB3	ENSG00000119699	7043
Hypertrophic cardiomyopathy	CACNG1	ENSG00000108878	786
Hypertrophic cardiomyopathy	ITGB6	ENSG00000115221	3694
Hypertrophic cardiomyopathy	TPM1	ENSG00000140416	7168
Hypertrophic cardiomyopathy	SGCG	ENSG00000102683	6445
Dilated cardiomyopathy	ITGA5	ENSG00000161638	3678
Dilated cardiomyopathy	TGFB3	ENSG00000119699	7043
Dilated cardiomyopathy	CACNG1	ENSG00000108878	786
Dilated cardiomyopathy	ITGB6	ENSG00000115221	3694
Dilated cardiomyopathy	TPM1	ENSG00000140416	7168
Dilated cardiomyopathy	SGCG	ENSG00000102683	6445
Arrhythmogenic right ventricula	ITGA5	ENSG00000161638	3678
Arrhythmogenic right ventricula	GJA1	ENSG00000152661	2697
Arrhythmogenic right ventricula	CACNG1	ENSG00000108878	786
Arrhythmogenic right ventricula	ITGB6	ENSG00000115221	3694
Arrhythmogenic right ventricula	SGCG	ENSG00000102683	6445
Yersinia infection	PIP5K1A	ENSG00000143398	8394
Yersinia infection	ITGA5	ENSG00000161638	3678
Yersinia infection	PIK3R3	ENSG00000117461	8503
Yersinia infection	CCL2	ENSG00000108691	6347
Long-term potentiation	BRAF	ENSG00000157764	673
Long-term potentiation	PPP3CA	ENSG00000138814	5530
Long-term potentiation	RAPGEF3	ENSG00000079337	10411
Gap junction	GJA1	ENSG00000152661	2697
Gap junction	GNAI1	ENSG00000127955	2770
Gap junction	TUBA4A	ENSG00000127824	7277
Phospholipase D signaling path	PIK3R3	ENSG00000117461	8503
Phospholipase D signaling path	PIP5K1A	ENSG00000143398	8394
Phospholipase D signaling path	SPHK2	ENSG00000063176	56848
Phospholipase D signaling path	RAPGEF3	ENSG00000079337	10411
Viral myocarditis	CD55	ENSG00000196352	1604
Viral myocarditis	ABL2	ENSG00000143322	27
Viral myocarditis	CAV1	ENSG00000105974	857
Viral myocarditis	SGCG	ENSG00000102683	6445
Long-term depression	BRAF	ENSG00000157764	673
Long-term depression	GNAI1	ENSG00000127955	2770
Antifolate resistance	ABCC1	ENSG00000103222	4363
Antifolate resistance	ABCC5	ENSG00000114770	10057
Antifolate resistance	MTHFR	ENSG00000177000	4524
Basal transcription factors	TAF2	ENSG00000064313	6873
IL-17 signaling pathway	FOSB	ENSG00000125740	2354
IL-17 signaling pathway	CXCL2	ENSG00000081041	2920
IL-17 signaling pathway	CCL2	ENSG00000108691	6347

Pertussis	ITGA5	ENSG00000161638	3678
Pertussis	GNAI1	ENSG00000127955	2770
Selenocompound metabolism	TXNRD1	ENSG00000198431	7296
Selenocompound metabolism	MARS2	ENSG00000247626	92935
Selenocompound metabolism	PAPSS1	ENSG00000138801	9061
Terpenoid backbone biosynthes	DHDDS	ENSG00000117682	79947
Terpenoid backbone biosynthes	MVD	ENSG00000167508	4597
Proteasome	PSME4	ENSG00000068878	23198
Morphine addiction	PDE4B	ENSG00000184588	5142
Morphine addiction	PDE4D	ENSG00000113448	5144
Morphine addiction	GNAI1	ENSG00000127955	2770
Morphine addiction	GNG11	ENSG00000127920	2791
Morphine addiction	PDE7A	ENSG00000205268	5150
Circadian entrainment	GNG11	ENSG00000127920	2791
Circadian entrainment	GNAI1	ENSG00000127955	2770
Circadian entrainment	PER3	ENSG00000049246	8863
Circadian entrainment	PER2	ENSG00000132326	8864
PPAR signaling pathway	CPT1A	ENSG00000110090	1374
PPAR signaling pathway	ACSL1	ENSG00000151726	2180
PPAR signaling pathway	ACSL3	ENSG00000123983	2181
PPAR signaling pathway	ACOX1	ENSG00000161533	51
PPAR signaling pathway	FABP4	ENSG00000170323	2167
PPAR signaling pathway	ACSL6	ENSG00000164398	23305
Leishmaniasis	TGFB3	ENSG00000119699	7043
Leishmaniasis	ELK1	ENSG00000126767	2002
Serotonergic synapse	BRAF	ENSG00000157764	673
Serotonergic synapse	GNAI1	ENSG00000127955	2770
Serotonergic synapse	GNG11	ENSG00000127920	2791
Serotonergic synapse	RAPGEF3	ENSG00000079337	10411
Glycolysis / Gluconeogenesis	HK2	ENSG00000159399	3099
Glycolysis / Gluconeogenesis	ADPGK	ENSG00000159322	83440
Glycolysis / Gluconeogenesis	ADH1B	ENSG00000196616	125
Glycolysis / Gluconeogenesis	GAPDH	ENSG00000111640	2597
Biosynthesis of amino acids	GAPDH	ENSG00000111640	2597
Biosynthesis of amino acids	ARG2	ENSG00000081181	384
Glucagon signaling pathway	CPT1A	ENSG00000110090	1374
Glucagon signaling pathway	ACACB	ENSG00000076555	32
Glucagon signaling pathway	PPP3CA	ENSG00000138814	5530
Cardiac muscle contraction	CACNG1	ENSG00000108878	786
Cardiac muscle contraction	TPM1	ENSG00000140416	7168
Fatty acid degradation	ACSL1	ENSG00000151726	2180
Fatty acid degradation	ACSL3	ENSG00000123983	2181
Fatty acid degradation	ACOX1	ENSG00000161533	51
Fatty acid degradation	CPT1A	ENSG00000110090	1374
Fatty acid degradation	ADH1B	ENSG00000196616	125
Fatty acid degradation	ACSL6	ENSG00000164398	23305
Glutamatergic synapse	SLC38A3	ENSG00000188338	10991
Glutamatergic synapse	GNAI1	ENSG00000127955	2770
Glutamatergic synapse	GNG11	ENSG00000127920	2791
Glutamatergic synapse	PPP3CA	ENSG00000138814	5530
Aldosterone synthesis and secr	NR4A2	ENSG00000153234	4929
Aldosterone synthesis and secr	LDLR	ENSG00000130164	3949
Aldosterone synthesis and secr	PRKCE	ENSG00000171132	5581
Propanoate metabolism	ACACB	ENSG00000076555	32
Propanoate metabolism	MLYCD	ENSG00000103150	23417
Propanoate metabolism	DBT	ENSG00000137992	1629
Propanoate metabolism	ACOX1	ENSG00000161533	51
GnRH signaling pathway	ELK1	ENSG00000126767	2002
Dopaminergic synapse	GNAI1	ENSG00000127955	2770

Dopaminergic synapse	GNG11	ENSG00000127920	2791
Dopaminergic synapse	PPP3CA	ENSG00000138814	5530

logFC	logCPM	F	PValue	FDR
0.6093	5.5716	23.8912	0.0001	0.0099
0.6691	3.6192	22.9722	0.0001	0.0114
1.3460	3.4650	50.0021	0.0000	0.0013
0.7415	4.0844	13.6770	0.0018	0.0404
0.3211	7.1475	12.8832	0.0021	0.0437
3.6753	4.5531	16.7786	0.0010	0.0299
3.9581	2.7826	29.8769	0.0000	0.0044
1.1543	3.7547	16.0412	0.0010	0.0299
2.0870	3.2489	39.1226	0.0000	0.0034
4.1234	5.1954	28.8053	0.0001	0.0082
-0.5015	2.1968	25.9154	0.0001	0.0082
0.3577	2.6804	18.6195	0.0004	0.0197
0.7415	4.0844	13.6770	0.0018	0.0404
0.3211	7.1475	12.8832	0.0021	0.0437
1.3460	3.4650	50.0021	0.0000	0.0013
1.0011	1.1062	16.5693	0.0007	0.0259
0.6093	5.5716	23.8912	0.0001	0.0099
0.9947	-0.0183	18.7395	0.0004	0.0197
0.4624	2.8334	20.6197	0.0003	0.0156
2.2055	4.6125	27.9647	0.0001	0.0082
0.6169	3.7040	32.7979	0.0000	0.0041
-0.5015	2.1968	25.9154	0.0001	0.0082
-0.6625	7.7608	25.5578	0.0001	0.0083
0.2887	4.2694	24.6984	0.0001	0.0087
1.3460	3.4650	50.0021	0.0000	0.0013
0.3912	3.3627	20.7318	0.0003	0.0153
0.3577	2.6804	18.6195	0.0004	0.0197
2.5425	5.3512	19.4088	0.0005	0.0219
2.2055	4.6125	27.9647	0.0001	0.0082
-0.7668	6.0634	47.6969	0.0000	0.0014
0.7109	3.6736	19.1794	0.0004	0.0192
2.5425	5.3512	19.4088	0.0005	0.0219
0.7415	4.0844	13.6770	0.0018	0.0404
0.6093	5.5716	23.8912	0.0001	0.0099
1.0631	3.7425	30.5231	0.0000	0.0056
1.3460	3.4650	50.0021	0.0000	0.0013
0.3594	2.9589	15.4734	0.0010	0.0299
0.2887	4.2694	24.6984	0.0001	0.0087
1.3846	5.9599	21.4943	0.0003	0.0158
3.9549	2.7136	20.3619	0.0002	0.0135
0.9534	0.7077	15.9589	0.0009	0.0284
0.3577	2.6804	18.6195	0.0004	0.0197
-0.3176	5.9710	12.8947	0.0021	0.0437
-0.3903	2.6115	13.2370	0.0019	0.0418
1.3460	3.4650	50.0021	0.0000	0.0013
3.6753	4.5531	16.7786	0.0010	0.0299
0.8538	4.1507	18.4253	0.0005	0.0211
2.5362	5.5931	52.5608	0.0000	0.0014
1.0418	2.3525	14.2629	0.0015	0.0370
0.3577	2.6804	18.6195	0.0004	0.0197
0.6093	5.5716	23.8912	0.0001	0.0099
1.0631	3.7425	30.5231	0.0000	0.0056
-0.6897	1.0753	15.7824	0.0009	0.0291
-0.2608	7.2955	14.4879	0.0013	0.0341
-0.4112	2.6413	13.0801	0.0020	0.0424
-0.5015	2.1968	25.9154	0.0001	0.0082
-0.5322	5.3994	17.8043	0.0005	0.0223
0.6082	0.0997	19.7829	0.0003	0.0176

1.2058	5.3895	30.9222	0.0000	0.0056
0.3677	5.9930	16.9824	0.0007	0.0247
1.1386	2.2396	13.2546	0.0021	0.0431
4.1234	5.1954	28.8053	0.0001	0.0082
2.5425	5.3512	19.4088	0.0005	0.0219
1.3460	3.4650	50.0021	0.0000	0.0013
1.0011	1.1062	16.5693	0.0007	0.0259
0.3482	3.8639	19.8194	0.0003	0.0174
-0.3225	4.9789	13.8958	0.0016	0.0379
0.3912	3.3627	20.7318	0.0003	0.0153
0.2887	4.2694	24.6984	0.0001	0.0087
1.3460	3.4650	50.0021	0.0000	0.0013
0.7109	3.6736	19.1794	0.0004	0.0192
2.5425	5.3512	19.4088	0.0005	0.0219
3.9549	2.7136	20.3619	0.0002	0.0135
0.3577	2.6804	18.6195	0.0004	0.0197
0.3618	6.9754	17.4297	0.0006	0.0231
0.3594	2.9589	15.4734	0.0010	0.0299
0.7109	3.6736	19.1794	0.0004	0.0192
1.3460	3.4650	50.0021	0.0000	0.0013
0.3912	3.3627	20.7318	0.0003	0.0153
0.3577	2.6804	18.6195	0.0004	0.0197
2.5425	5.3512	19.4088	0.0005	0.0219
2.2055	4.6125	27.9647	0.0001	0.0082
0.9947	-0.0183	18.7395	0.0004	0.0197
0.6169	3.7040	32.7979	0.0000	0.0041
-0.2864	6.0658	13.0037	0.0020	0.0428
0.4602	2.1437	29.5337	0.0000	0.0056
0.9842	2.3978	32.2303	0.0000	0.0044
2.5425	5.3512	19.4088	0.0005	0.0219
1.2323	2.5083	17.9076	0.0006	0.0230
0.3612	8.0741	14.5054	0.0013	0.0341
2.2188	4.8714	21.2799	0.0003	0.0181
1.9788	2.7562	19.8128	0.0004	0.0195
0.9882	4.3803	17.5518	0.0006	0.0247
0.3675	2.6915	23.1324	0.0001	0.0112
0.3577	2.6804	18.6195	0.0004	0.0197
2.2055	4.6125	27.9647	0.0001	0.0082
-0.3964	2.4077	15.6494	0.0009	0.0296
-0.5656	5.9475	34.6539	0.0000	0.0040
-0.7418	4.1843	17.2233	0.0007	0.0247
-0.3889	2.5857	13.2973	0.0019	0.0413
-0.3626	2.7118	14.5745	0.0013	0.0337
-0.4112	2.6413	13.0801	0.0020	0.0424
0.2732	3.6375	12.8977	0.0021	0.0437
0.2595	3.1601	13.1356	0.0020	0.0421
0.4008	4.6795	24.4083	0.0001	0.0091
0.7867	2.8742	15.6469	0.0010	0.0299
-0.2919	3.6510	20.7294	0.0003	0.0153
0.8210	6.0710	15.5246	0.0011	0.0309
0.5367	4.9082	14.1218	0.0015	0.0369
0.2743	4.5520	13.3779	0.0018	0.0411
2.3373	0.9517	27.1856	0.0000	0.0061
0.7415	4.0844	13.6770	0.0018	0.0404
0.3912	3.3627	20.7318	0.0003	0.0153
0.3577	2.6804	18.6195	0.0004	0.0197
0.2887	4.2694	24.6984	0.0001	0.0087
2.2055	4.6125	27.9647	0.0001	0.0082
1.0631	3.7425	30.5231	0.0000	0.0056

0.6093	5.5716	23.8912	0.0001	0.0099
0.7109	3.6736	19.1794	0.0004	0.0192
1.1543	3.7547	16.0412	0.0010	0.0299
-0.5604	4.4847	15.0868	0.0011	0.0316
-0.2517	7.5526	13.5754	0.0017	0.0399
-0.5015	2.1968	25.9154	0.0001	0.0082
-0.7318	1.5868	12.6570	0.0023	0.0458
-0.3176	5.9710	12.8947	0.0021	0.0437
-0.4344	3.9918	12.7219	0.0022	0.0451
1.3460	3.4650	50.0021	0.0000	0.0013
0.3594	2.9589	15.4734	0.0010	0.0299
1.1559	2.8092	13.2461	0.0021	0.0440
0.3912	3.3627	20.7318	0.0003	0.0153
0.3577	2.6804	18.6195	0.0004	0.0197
2.5425	5.3512	19.4088	0.0005	0.0219
2.2055	4.6125	27.9647	0.0001	0.0082
-0.7668	6.0634	47.6969	0.0000	0.0014
1.3460	3.4650	50.0021	0.0000	0.0013
0.3912	3.3627	20.7318	0.0003	0.0153
0.3594	2.9589	15.4734	0.0010	0.0299
0.7109	3.6736	19.1794	0.0004	0.0192
0.3577	2.6804	18.6195	0.0004	0.0197
2.5425	5.3512	19.4088	0.0005	0.0219
2.2055	4.6125	27.9647	0.0001	0.0082
1.1559	2.8092	13.2461	0.0021	0.0440
0.3577	2.6804	18.6195	0.0004	0.0197
1.3460	3.4650	50.0021	0.0000	0.0013
2.5425	5.3512	19.4088	0.0005	0.0219
0.3594	2.9589	15.4734	0.0010	0.0299
2.2055	4.6125	27.9647	0.0001	0.0082
0.9132	5.4611	19.5200	0.0004	0.0192
0.5210	4.7637	19.6602	0.0003	0.0178
0.7109	3.6736	19.1794	0.0004	0.0192
-0.7668	6.0634	47.6969	0.0000	0.0014
-0.3176	5.9710	12.8947	0.0021	0.0437
1.3460	3.4650	50.0021	0.0000	0.0013
0.6093	5.5716	23.8912	0.0001	0.0099
0.3677	5.9930	16.9824	0.0007	0.0247
2.5425	5.3512	19.4088	0.0005	0.0219
0.3577	2.6804	18.6195	0.0004	0.0197
0.3594	2.9589	15.4734	0.0010	0.0299
1.6279	-0.2524	35.9301	0.0000	0.0033
0.2391	4.3802	12.5866	0.0023	0.0464
3.9581	2.7826	29.8769	0.0000	0.0044
-0.4567	3.5603	13.9638	0.0015	0.0375
-0.3176	5.9710	12.8947	0.0021	0.0437
-0.3451	4.7455	14.7714	0.0012	0.0330
2.5425	5.3512	19.4088	0.0005	0.0219
0.3594	2.9589	15.4734	0.0010	0.0299
0.3577	2.6804	18.6195	0.0004	0.0197
1.3460	3.4650	50.0021	0.0000	0.0013
0.3912	3.3627	20.7318	0.0003	0.0153
2.2188	4.8714	21.2799	0.0003	0.0181
-0.4112	2.6413	13.0801	0.0020	0.0424
1.6386	3.8118	28.4323	0.0001	0.0075
-0.9123	5.2655	75.5953	0.0000	0.0003
-0.7418	4.1843	17.2233	0.0007	0.0247
-0.9984	3.8611	32.8188	0.0000	0.0043
-1.8747	4.9824	77.2658	0.0000	0.0003

-0.7787	5.7761	46.6006	0.0000	0.0014
1.3460	3.4650	50.0021	0.0000	0.0013
0.6093	5.5716	23.8912	0.0001	0.0099
3.6753	4.5531	16.7786	0.0010	0.0299
1.2058	5.3895	30.9222	0.0000	0.0056
1.0631	3.7425	30.5231	0.0000	0.0056
1.6279	-0.2524	35.9301	0.0000	0.0033
2.5425	5.3512	19.4088	0.0005	0.0219
0.3577	2.6804	18.6195	0.0004	0.0197
0.3594	2.9589	15.4734	0.0010	0.0299
0.2887	4.2694	24.6984	0.0001	0.0087
-0.4567	3.5603	13.9638	0.0015	0.0375
-0.3176	5.9710	12.8947	0.0021	0.0437
-0.3873	2.3535	15.8797	0.0009	0.0286
1.9788	2.7562	19.8128	0.0004	0.0195
0.3577	2.6804	18.6195	0.0004	0.0197
0.7109	3.6736	19.1794	0.0004	0.0192
2.5425	5.3512	19.4088	0.0005	0.0219
0.3594	2.9589	15.4734	0.0010	0.0299
1.3460	3.4650	50.0021	0.0000	0.0013
0.3912	3.3627	20.7318	0.0003	0.0153
2.2055	4.6125	27.9647	0.0001	0.0082
0.6741	4.5405	16.1855	0.0008	0.0281
4.1234	5.1954	28.8053	0.0001	0.0082
0.3912	3.3627	20.7318	0.0003	0.0153
1.3460	3.4650	50.0021	0.0000	0.0013
0.3577	2.6804	18.6195	0.0004	0.0197
2.5425	5.3512	19.4088	0.0005	0.0219
0.6093	5.5716	23.8912	0.0001	0.0099
1.6255	2.8542	14.6589	0.0015	0.0368
0.5975	2.4916	26.6277	0.0001	0.0076
0.7791	1.7234	17.8251	0.0005	0.0222
0.3594	2.9589	15.4734	0.0010	0.0299
0.3211	7.1475	12.8832	0.0021	0.0437
0.3912	3.3627	20.7318	0.0003	0.0153
1.3460	3.4650	50.0021	0.0000	0.0013
0.5332	3.5605	17.9491	0.0005	0.0218
0.3577	2.6804	18.6195	0.0004	0.0197
2.5425	5.3512	19.4088	0.0005	0.0219
0.3594	2.9589	15.4734	0.0010	0.0299
0.7084	2.0394	16.1266	0.0008	0.0278
1.3460	3.4650	50.0021	0.0000	0.0013
0.3577	2.6804	18.6195	0.0004	0.0197
1.0418	2.3525	14.2629	0.0015	0.0370
3.6753	4.5531	16.7786	0.0010	0.0299
0.7312	4.6298	35.5110	0.0000	0.0037
0.4624	2.8334	20.6197	0.0003	0.0156
0.4643	3.9560	42.7186	0.0000	0.0018
3.9581	2.7826	29.8769	0.0000	0.0044
0.6093	5.5716	23.8912	0.0001	0.0099
0.4331	3.4711	32.7228	0.0000	0.0041
1.6255	2.8542	14.6589	0.0015	0.0368
-0.5015	2.1968	25.9154	0.0001	0.0082
-0.3176	5.9710	12.8947	0.0021	0.0437
-0.7318	1.5868	12.6570	0.0023	0.0458
0.3577	2.6804	18.6195	0.0004	0.0197
0.2391	4.3802	12.5866	0.0023	0.0464
2.5362	5.5931	52.5608	0.0000	0.0014
0.3747	8.0026	21.5331	0.0002	0.0141

0.3984	5.5924	32.7891	0.0000	0.0041
0.4288	5.0635	21.3444	0.0002	0.0145
-0.5072	2.1776	14.4357	0.0013	0.0345
0.3912	3.3627	20.7318	0.0003	0.0153
0.3577	2.6804	18.6195	0.0004	0.0197
2.5425	5.3512	19.4088	0.0005	0.0219
2.2055	4.6125	27.9647	0.0001	0.0082
1.3460	3.4650	50.0021	0.0000	0.0013
0.5980	2.6344	33.6618	0.0000	0.0041
0.4881	5.5673	19.3232	0.0004	0.0190
0.3577	2.6804	18.6195	0.0004	0.0197
1.0418	2.3525	14.2629	0.0015	0.0370
0.6093	5.5716	23.8912	0.0001	0.0099
-0.3873	2.3535	15.8797	0.0009	0.0286
-0.5015	2.1968	25.9154	0.0001	0.0082
-0.6463	2.5018	44.6529	0.0000	0.0016
1.3460	3.4650	50.0021	0.0000	0.0013
0.3912	3.3627	20.7318	0.0003	0.0153
0.3577	2.6804	18.6195	0.0004	0.0197
0.5081	3.6359	20.0730	0.0003	0.0168
0.3813	3.6268	12.5430	0.0024	0.0467
0.2768	4.1864	16.0130	0.0008	0.0281
-0.7668	6.0634	47.6969	0.0000	0.0014
-0.5015	2.1968	25.9154	0.0001	0.0082
0.3912	3.3627	20.7318	0.0003	0.0153
1.3460	3.4650	50.0021	0.0000	0.0013
2.5425	5.3512	19.4088	0.0005	0.0219
0.7325	2.5212	19.9238	0.0003	0.0170
2.2055	4.6125	27.9647	0.0001	0.0082
0.6169	3.7040	32.7979	0.0000	0.0041
0.8538	4.1507	18.4253	0.0005	0.0211
0.5569	6.6421	20.2848	0.0003	0.0163
1.0351	6.7272	15.2373	0.0012	0.0331
0.7109	3.6736	19.1794	0.0004	0.0192
-0.7668	6.0634	47.6969	0.0000	0.0014
0.2893	5.5537	15.6256	0.0009	0.0296
0.3912	3.3627	20.7318	0.0003	0.0153
0.5332	3.5605	17.9491	0.0005	0.0218
0.7084	2.0394	16.1266	0.0008	0.0278
1.3460	3.4650	50.0021	0.0000	0.0013
0.6269	2.3660	15.0734	0.0011	0.0316
0.3594	2.9589	15.4734	0.0010	0.0299
2.5425	5.3512	19.4088	0.0005	0.0219
0.6070	2.3683	13.2338	0.0019	0.0418
0.3577	2.6804	18.6195	0.0004	0.0197
2.2055	4.6125	27.9647	0.0001	0.0082
0.3577	2.6804	18.6195	0.0004	0.0197
0.9947	-0.0183	18.7395	0.0004	0.0197
1.3460	3.4650	50.0021	0.0000	0.0013
3.9549	2.7136	20.3619	0.0002	0.0135
2.5425	5.3512	19.4088	0.0005	0.0219
0.8320	6.1915	35.8840	0.0000	0.0035
0.3677	5.9930	16.9824	0.0007	0.0247
0.3618	6.9754	17.4297	0.0006	0.0231
-1.1916	3.5629	49.5312	0.0000	0.0013
4.1234	5.1954	28.8053	0.0001	0.0082
1.3460	3.4650	50.0021	0.0000	0.0013
0.6169	3.7040	32.7979	0.0000	0.0041
0.4624	2.8334	20.6197	0.0003	0.0156

1.2058	5.3895	30.9222	0.0000	0.0056
0.6093	5.5716	23.8912	0.0001	0.0099
-0.5015	2.1968	25.9154	0.0001	0.0082
-0.2794	6.7890	13.4386	0.0018	0.0407
0.3912	3.3627	20.7318	0.0003	0.0153
1.3460	3.4650	50.0021	0.0000	0.0013
0.3577	2.6804	18.6195	0.0004	0.0197
0.3594	2.9589	15.4734	0.0010	0.0299
2.5425	5.3512	19.4088	0.0005	0.0219
2.2055	4.6125	27.9647	0.0001	0.0082
0.3912	3.3627	20.7318	0.0003	0.0153
2.5425	5.3512	19.4088	0.0005	0.0219
0.3577	2.6804	18.6195	0.0004	0.0197
0.3594	2.9589	15.4734	0.0010	0.0299
1.3460	3.4650	50.0021	0.0000	0.0013
2.2055	4.6125	27.9647	0.0001	0.0082
1.3460	3.4650	50.0021	0.0000	0.0013
0.3912	3.3627	20.7318	0.0003	0.0153
0.6169	3.7040	32.7979	0.0000	0.0041
1.2058	5.3895	30.9222	0.0000	0.0056
0.8210	0.9289	17.0587	0.0006	0.0229
-0.7668	6.0634	47.6969	0.0000	0.0014
0.3577	2.6804	18.6195	0.0004	0.0197
0.9947	-0.0183	18.7395	0.0004	0.0197
1.3460	3.4650	50.0021	0.0000	0.0013
2.5425	5.3512	19.4088	0.0005	0.0219
-0.6897	1.0753	15.7824	0.0009	0.0291
-0.2608	7.2955	14.4879	0.0013	0.0341
-0.5015	2.1968	25.9154	0.0001	0.0082
1.0631	3.7425	30.5231	0.0000	0.0056
0.6093	5.5716	23.8912	0.0001	0.0099
0.7415	4.0844	13.6770	0.0018	0.0404
2.2188	4.8714	21.2799	0.0003	0.0181
0.3211	7.1475	12.8832	0.0021	0.0437
2.2055	4.6125	27.9647	0.0001	0.0082
3.9581	2.7826	29.8769	0.0000	0.0044
3.9581	2.7826	29.8769	0.0000	0.0044
1.0631	3.7425	30.5231	0.0000	0.0056
1.3460	3.4650	50.0021	0.0000	0.0013
0.7109	3.6736	19.1794	0.0004	0.0192
-1.0513	3.7214	29.1514	0.0000	0.0060
0.3577	2.6804	18.6195	0.0004	0.0197
0.7415	4.0844	13.6770	0.0018	0.0404
1.3460	3.4650	50.0021	0.0000	0.0013
2.5425	5.3512	19.4088	0.0005	0.0219
2.2055	4.6125	27.9647	0.0001	0.0082
0.3594	2.9589	15.4734	0.0010	0.0299
0.6169	3.7040	32.7979	0.0000	0.0041
1.3460	3.4650	50.0021	0.0000	0.0013
0.3594	2.9589	15.4734	0.0010	0.0299
0.3577	2.6804	18.6195	0.0004	0.0197
2.5425	5.3512	19.4088	0.0005	0.0219
2.2055	4.6125	27.9647	0.0001	0.0082
1.3460	3.4650	50.0021	0.0000	0.0013
0.3577	2.6804	18.6195	0.0004	0.0197
-0.7668	6.0634	47.6969	0.0000	0.0014
-0.4835	7.1267	24.5692	0.0001	0.0089
-0.2864	6.0658	13.0037	0.0020	0.0428
1.1673	1.9510	13.7783	0.0017	0.0399

0.8320	6.1915	35.8840	0.0000	0.0035
2.5362	5.5931	52.5608	0.0000	0.0014
1.3460	3.4650	50.0021	0.0000	0.0013
0.3594	2.9589	15.4734	0.0010	0.0299
0.5332	3.5605	17.9491	0.0005	0.0218
0.6169	3.7040	32.7979	0.0000	0.0041
0.8225	-0.4212	13.0012	0.0020	0.0430
2.5425	5.3512	19.4088	0.0005	0.0219
0.7993	2.4639	13.9801	0.0016	0.0379
0.4873	4.0931	12.5508	0.0023	0.0467
2.2188	4.8714	21.2799	0.0003	0.0181
0.4881	5.5673	19.3232	0.0004	0.0190
0.3577	2.6804	18.6195	0.0004	0.0197
1.2736	2.3222	37.3660	0.0000	0.0033
-0.2664	11.7779	17.5248	0.0006	0.0229
-0.6463	2.5018	44.6529	0.0000	0.0016
0.7069	3.1144	29.1881	0.0000	0.0056
0.4454	7.2762	15.3787	0.0010	0.0302
0.5761	5.6917	15.2554	0.0011	0.0309
0.6093	5.5716	23.8912	0.0001	0.0099
0.7415	4.0844	13.6770	0.0018	0.0404
1.3908	0.3673	19.2529	0.0004	0.0192
3.9581	2.7826	29.8769	0.0000	0.0044
0.4258	7.3496	26.8295	0.0001	0.0075
0.2501	5.5907	17.8782	0.0005	0.0220
-0.7668	6.0634	47.6969	0.0000	0.0014
-0.5015	2.1968	25.9154	0.0001	0.0082
-0.6731	5.0064	34.3066	0.0000	0.0041
0.3912	3.3627	20.7318	0.0003	0.0153
1.3460	3.4650	50.0021	0.0000	0.0013
0.6269	2.3660	15.0734	0.0011	0.0316
0.3594	2.9589	15.4734	0.0010	0.0299
0.3577	2.6804	18.6195	0.0004	0.0197
2.5425	5.3512	19.4088	0.0005	0.0219
2.2055	4.6125	27.9647	0.0001	0.0082
0.2887	4.2694	24.6984	0.0001	0.0087
0.7109	3.6736	19.1794	0.0004	0.0192
2.5362	5.5931	52.5608	0.0000	0.0014
0.4190	5.7579	34.0132	0.0000	0.0041
-0.6897	1.0753	15.7824	0.0009	0.0291
-0.2608	7.2955	14.4879	0.0013	0.0341
0.8380	1.9148	37.7021	0.0000	0.0033
0.3732	3.4965	14.9209	0.0012	0.0323
0.6006	3.3361	14.1522	0.0014	0.0365
0.8376	0.8685	16.8473	0.0006	0.0234
1.0732	4.5938	20.7151	0.0003	0.0169
1.0011	2.7642	17.9749	0.0005	0.0227
0.8832	2.9593	44.0701	0.0000	0.0016
0.8598	3.4348	33.2845	0.0000	0.0041
1.3460	3.4650	50.0021	0.0000	0.0013
-0.3873	2.3535	15.8797	0.0009	0.0286
-0.3176	5.9710	12.8947	0.0021	0.0437
-0.7683	2.5372	53.0223	0.0000	0.0011
-0.4587	1.8712	14.8545	0.0012	0.0327
4.1234	5.1954	28.8053	0.0001	0.0082
0.7069	3.1144	29.1881	0.0000	0.0056
0.3747	8.0026	21.5331	0.0002	0.0141
0.3984	5.5924	32.7891	0.0000	0.0041
0.4454	7.2762	15.3787	0.0010	0.0302

0.6691	3.6192	22.9722	0.0001	0.0114
0.6093	5.5716	23.8912	0.0001	0.0099
-0.5072	2.1776	14.4357	0.0013	0.0345
-0.2864	6.0658	13.0037	0.0020	0.0428
0.2887	4.2694	24.6984	0.0001	0.0087
2.5425	5.3512	19.4088	0.0005	0.0219
0.8210	0.9289	17.0587	0.0006	0.0229
0.6195	2.9529	15.3370	0.0010	0.0304
1.3460	3.4650	50.0021	0.0000	0.0013
0.3912	3.3627	20.7318	0.0003	0.0153
1.3460	3.4650	50.0021	0.0000	0.0013
0.2887	4.2694	24.6984	0.0001	0.0087
0.3912	3.3627	20.7318	0.0003	0.0153
0.3612	8.0741	14.5054	0.0013	0.0341
0.3577	2.6804	18.6195	0.0004	0.0197
2.2188	4.8714	21.2799	0.0003	0.0181
2.0169	1.3312	15.5315	0.0012	0.0323
2.5425	5.3512	19.4088	0.0005	0.0219
0.6269	2.3660	15.0734	0.0011	0.0316
0.4873	4.0931	12.5508	0.0023	0.0467
-0.5322	5.3994	17.8043	0.0005	0.0223
0.6093	5.5716	23.8912	0.0001	0.0099
1.0631	3.7425	30.5231	0.0000	0.0056
0.2391	4.3802	12.5866	0.0023	0.0464
0.4873	4.0931	12.5508	0.0023	0.0467
1.3460	3.4650	50.0021	0.0000	0.0013
0.3577	2.6804	18.6195	0.0004	0.0197
0.6381	5.0308	14.1739	0.0015	0.0371
-0.6463	2.5018	44.6529	0.0000	0.0016
-0.3451	4.7455	14.7714	0.0012	0.0330
-0.7668	6.0634	47.6969	0.0000	0.0014
1.3460	3.4650	50.0021	0.0000	0.0013
0.3912	3.3627	20.7318	0.0003	0.0153
0.7415	4.0844	13.6770	0.0018	0.0404
0.6195	2.9529	15.3370	0.0010	0.0304
1.1673	1.9510	13.7783	0.0017	0.0399
2.5362	5.5931	52.5608	0.0000	0.0014
-0.2794	6.7890	13.4386	0.0018	0.0407
-0.7668	6.0634	47.6969	0.0000	0.0014
-0.5015	2.1968	25.9154	0.0001	0.0082
-0.6897	1.0753	15.7824	0.0009	0.0291
-0.2608	7.2955	14.4879	0.0013	0.0341
0.9882	4.3803	17.5518	0.0006	0.0247
1.9788	2.7562	19.8128	0.0004	0.0195
1.3460	3.4650	50.0021	0.0000	0.0013
0.3912	3.3627	20.7318	0.0003	0.0153
-0.7418	4.1843	17.2233	0.0007	0.0247
1.1543	3.7547	16.0412	0.0010	0.0299
0.7415	4.0844	13.6770	0.0018	0.0404
1.3460	3.4650	50.0021	0.0000	0.0013
1.0631	3.7425	30.5231	0.0000	0.0056
4.6051	4.6138	18.0442	0.0004	0.0201
1.1438	5.8934	18.9414	0.0005	0.0210
0.6093	5.5716	23.8912	0.0001	0.0099
4.1234	5.1954	28.8053	0.0001	0.0082
-0.3176	5.9710	12.8947	0.0021	0.0437
1.3460	3.4650	50.0021	0.0000	0.0013
0.6381	5.0308	14.1739	0.0015	0.0371
2.1867	9.1171	15.1248	0.0014	0.0366

1.0418	2.3525	14.2629	0.0015	0.0370
2.5362	5.5931	52.5608	0.0000	0.0014
1.2058	5.3895	30.9222	0.0000	0.0056
2.5425	5.3512	19.4088	0.0005	0.0219
-0.2550	13.6189	5.8762	0.0262	0.1573
-0.4344	3.9918	12.7219	0.0022	0.0451
0.4873	4.0931	12.5508	0.0023	0.0467
1.3460	3.4650	50.0021	0.0000	0.0013
0.2915	5.9320	22.7570	0.0002	0.0117
-0.5322	5.3994	17.8043	0.0005	0.0223
2.2055	4.6125	27.9647	0.0001	0.0082
2.5425	5.3512	19.4088	0.0005	0.0219
0.3577	2.6804	18.6195	0.0004	0.0197
0.9947	-0.0183	18.7395	0.0004	0.0197
-0.4835	7.1267	24.5692	0.0001	0.0089
0.2687	3.5465	13.6076	0.0017	0.0397
-0.3673	3.7518	14.7040	0.0012	0.0331
2.5425	5.3512	19.4088	0.0005	0.0219
0.3577	2.6804	18.6195	0.0004	0.0197
0.3594	2.9589	15.4734	0.0010	0.0299
0.3912	3.3627	20.7318	0.0003	0.0153
0.4873	4.0931	12.5508	0.0023	0.0467
0.5775	2.7136	12.4464	0.0024	0.0474
1.3460	3.4650	50.0021	0.0000	0.0013
0.3369	5.0913	13.4289	0.0018	0.0407
0.2887	4.2694	24.6984	0.0001	0.0087
0.3912	3.3627	20.7318	0.0003	0.0153
-0.3458	6.1748	18.5498	0.0004	0.0198
-0.5322	5.3994	17.8043	0.0005	0.0223
1.2058	5.3895	30.9222	0.0000	0.0056
0.5975	2.4916	26.6277	0.0001	0.0076
0.7791	1.7234	17.8251	0.0005	0.0222
0.3414	5.2054	18.7713	0.0004	0.0196
3.6753	4.5531	16.7786	0.0010	0.0299
0.6093	5.5716	23.8912	0.0001	0.0099
0.3677	5.9930	16.9824	0.0007	0.0247
1.3908	0.3673	19.2529	0.0004	0.0192
1.3460	3.4650	50.0021	0.0000	0.0013
-0.2585	5.6083	13.8001	0.0016	0.0384
-0.2955	5.8208	16.2255	0.0008	0.0273
-0.3451	4.7455	14.7714	0.0012	0.0330
-0.4578	2.6694	13.3113	0.0019	0.0413
-0.2543	7.7794	13.1148	0.0020	0.0422
-0.2923	6.3057	14.6885	0.0012	0.0331
0.2723	5.7446	13.5711	0.0017	0.0399
0.8210	6.0710	15.5246	0.0011	0.0309
0.3577	2.6804	18.6195	0.0004	0.0197
0.2915	5.9320	22.7570	0.0002	0.0117
0.4624	2.8334	20.6197	0.0003	0.0156
-0.2794	6.7890	13.4386	0.0018	0.0407
-0.5015	2.1968	25.9154	0.0001	0.0082
-0.6625	7.7608	25.5578	0.0001	0.0083
0.6269	2.3660	15.0734	0.0011	0.0316
0.3677	5.9930	16.9824	0.0007	0.0247
1.2364	2.8412	23.6961	0.0001	0.0112
1.3460	3.4650	50.0021	0.0000	0.0013
0.7622	3.3671	62.4433	0.0000	0.0006
0.9842	2.3978	32.2303	0.0000	0.0044
0.5453	3.1136	20.8432	0.0002	0.0152

0.5328	4.5385	24.9896	0.0001	0.0085
-0.3225	4.9789	13.8958	0.0016	0.0379
-0.3626	2.7118	14.5745	0.0013	0.0337
0.3912	3.3627	20.7318	0.0003	0.0153
1.3460	3.4650	50.0021	0.0000	0.0013
0.6269	2.3660	15.0734	0.0011	0.0316
0.3577	2.6804	18.6195	0.0004	0.0197
2.5425	5.3512	19.4088	0.0005	0.0219
2.2055	4.6125	27.9647	0.0001	0.0082
0.7109	3.6736	19.1794	0.0004	0.0192
0.3594	2.9589	15.4734	0.0010	0.0299
-0.2955	5.8208	16.2255	0.0008	0.0273
-0.3451	4.7455	14.7714	0.0012	0.0330
-0.4578	2.6694	13.3113	0.0019	0.0413
-0.2585	5.6083	13.8001	0.0016	0.0384
-0.2923	6.3057	14.6885	0.0012	0.0331
-0.2543	7.7794	13.1148	0.0020	0.0422
-0.3969	4.0709	21.2034	0.0002	0.0148
1.7505	5.7691	26.5695	0.0001	0.0085
0.5697	6.3701	87.8065	0.0000	0.0002
0.3912	3.3627	20.7318	0.0003	0.0153
2.5425	5.3512	19.4088	0.0005	0.0219
1.7061	2.2893	15.2344	0.0012	0.0331
1.0351	6.7272	15.2373	0.0012	0.0331
-0.3873	2.3535	15.8797	0.0009	0.0286
0.5980	2.6344	33.6618	0.0000	0.0041
1.3460	3.4650	50.0021	0.0000	0.0013
1.0418	2.3525	14.2629	0.0015	0.0370
-0.3176	5.9710	12.8947	0.0021	0.0437
0.5775	2.7136	12.4464	0.0024	0.0474
1.0418	2.3525	14.2629	0.0015	0.0370
-0.3176	5.9710	12.8947	0.0021	0.0437
-0.6463	2.5018	44.6529	0.0000	0.0016
-0.3873	2.3535	15.8797	0.0009	0.0286
0.6269	2.3660	15.0734	0.0011	0.0316
0.3577	2.6804	18.6195	0.0004	0.0197
2.5425	5.3512	19.4088	0.0005	0.0219
2.2055	4.6125	27.9647	0.0001	0.0082
0.7109	3.6736	19.1794	0.0004	0.0192
1.3460	3.4650	50.0021	0.0000	0.0013
0.3912	3.3627	20.7318	0.0003	0.0153
2.5425	5.3512	19.4088	0.0005	0.0219
0.5975	2.4916	26.6277	0.0001	0.0076
0.7791	1.7234	17.8251	0.0005	0.0222
0.4271	5.1130	28.8137	0.0000	0.0058
0.3278	3.1712	17.1394	0.0006	0.0242
-0.3943	5.1781	39.5528	0.0000	0.0025
-0.3889	2.5857	13.2973	0.0019	0.0413
1.3460	3.4650	50.0021	0.0000	0.0013
0.4624	2.8334	20.6197	0.0003	0.0156
0.3211	7.1475	12.8832	0.0021	0.0437
0.5569	6.6421	20.2848	0.0003	0.0163
0.2391	4.3802	12.5866	0.0023	0.0464
0.3829	3.8751	16.4089	0.0008	0.0265
0.4410	3.7555	14.5708	0.0013	0.0337
-0.6054	3.7801	17.6843	0.0005	0.0227
-0.3451	4.7455	14.7714	0.0012	0.0330
0.4454	7.2762	15.3787	0.0010	0.0302
0.3747	8.0026	21.5331	0.0002	0.0141

0.3984	5.5924	32.7891	0.0000	0.0041
-0.5072	2.1776	14.4357	0.0013	0.0345
1.6279	-0.2524	35.9301	0.0000	0.0033
3.6753	4.5531	16.7786	0.0010	0.0299
3.9581	2.7826	29.8769	0.0000	0.0044
0.6123	4.0184	18.0834	0.0005	0.0213
0.3677	5.9930	16.9824	0.0007	0.0247
1.2058	5.3895	30.9222	0.0000	0.0056
1.1543	3.7547	16.0412	0.0010	0.0299
0.6093	5.5716	23.8912	0.0001	0.0099
0.6691	3.6192	22.9722	0.0001	0.0114
0.9091	0.7168	13.6473	0.0017	0.0395
1.0631	3.7425	30.5231	0.0000	0.0056
1.3460	3.4650	50.0021	0.0000	0.0013
-0.6463	2.5018	44.6529	0.0000	0.0016
0.7109	3.6736	19.1794	0.0004	0.0192
1.3460	3.4650	50.0021	0.0000	0.0013
1.0418	2.3525	14.2629	0.0015	0.0370
-0.2794	6.7890	13.4386	0.0018	0.0407
-0.2550	13.6189	5.8762	0.0262	0.1573
1.0011	1.1062	16.5693	0.0007	0.0259
0.3677	5.9930	16.9824	0.0007	0.0247
1.0631	3.7425	30.5231	0.0000	0.0056
0.7109	3.6736	19.1794	0.0004	0.0192
0.6082	0.0997	19.7829	0.0003	0.0176
1.1386	2.2396	13.2546	0.0021	0.0431
1.2058	5.3895	30.9222	0.0000	0.0056
0.6093	5.5716	23.8912	0.0001	0.0099
0.6691	3.6192	22.9722	0.0001	0.0114
0.9091	0.7168	13.6473	0.0017	0.0395
3.9581	2.7826	29.8769	0.0000	0.0044
0.6123	4.0184	18.0834	0.0005	0.0213
1.6279	-0.2524	35.9301	0.0000	0.0033
3.6753	4.5531	16.7786	0.0010	0.0299
1.1543	3.7547	16.0412	0.0010	0.0299
-0.3225	4.9789	13.8958	0.0016	0.0379
0.6093	5.5716	23.8912	0.0001	0.0099
1.3460	3.4650	50.0021	0.0000	0.0013
0.7109	3.6736	19.1794	0.0004	0.0192
0.3211	7.1475	12.8832	0.0021	0.0437
3.6753	4.5531	16.7786	0.0010	0.0299
-0.3873	2.3535	15.8797	0.0009	0.0286
0.6093	5.5716	23.8912	0.0001	0.0099
1.3460	3.4650	50.0021	0.0000	0.0013
4.1234	5.1954	28.8053	0.0001	0.0082
0.5704	7.6191	13.4888	0.0018	0.0408
1.0418	2.3525	14.2629	0.0015	0.0370
0.4454	7.2762	15.3787	0.0010	0.0302
0.7069	3.1144	29.1881	0.0000	0.0056
-0.6463	2.5018	44.6529	0.0000	0.0016
1.3460	3.4650	50.0021	0.0000	0.0013
4.1234	5.1954	28.8053	0.0001	0.0082
-0.7668	6.0634	47.6969	0.0000	0.0014
1.0631	3.7425	30.5231	0.0000	0.0056
1.2058	5.3895	30.9222	0.0000	0.0056
0.3677	5.9930	16.9824	0.0007	0.0247
1.9788	2.7562	19.8128	0.0004	0.0195
0.9882	4.3803	17.5518	0.0006	0.0247
-0.3176	5.9710	12.8947	0.0021	0.0437

0.6741	4.5405	16.1855	0.0008	0.0281
0.6093	5.5716	23.8912	0.0001	0.0099
1.0631	3.7425	30.5231	0.0000	0.0056
0.6735	3.0432	20.0306	0.0003	0.0169
-0.6625	7.7608	25.5578	0.0001	0.0083
-0.2550	13.6189	5.8762	0.0262	0.1573
2.5425	5.3512	19.4088	0.0005	0.0219
2.2055	4.6125	27.9647	0.0001	0.0082
0.3577	2.6804	18.6195	0.0004	0.0197
0.7109	3.6736	19.1794	0.0004	0.0192
0.3594	2.9589	15.4734	0.0010	0.0299
0.8225	-0.4212	13.0012	0.0020	0.0430
-0.3903	2.6115	13.2370	0.0019	0.0418
4.6051	4.6138	18.0442	0.0004	0.0201
-0.3176	5.9710	12.8947	0.0021	0.0437
1.6255	2.8542	14.6589	0.0015	0.0368
0.5552	3.0045	36.6635	0.0000	0.0033
0.6269	2.3660	15.0734	0.0011	0.0316
2.5425	5.3512	19.4088	0.0005	0.0219
0.3594	2.9589	15.4734	0.0010	0.0299
0.2976	3.3633	13.6932	0.0017	0.0392
0.3912	3.3627	20.7318	0.0003	0.0153
-0.3873	2.3535	15.8797	0.0009	0.0286
0.5569	6.6421	20.2848	0.0003	0.0163
-0.3873	2.3535	15.8797	0.0009	0.0286
-0.4567	3.5603	13.9638	0.0015	0.0375
-0.4400	5.6372	19.9640	0.0003	0.0169
1.3460	3.4650	50.0021	0.0000	0.0013
1.0418	2.3525	14.2629	0.0015	0.0370
0.2893	5.5537	15.6256	0.0009	0.0296
0.9882	4.3803	17.5518	0.0006	0.0247
-0.7318	1.5868	12.6570	0.0023	0.0458
-0.3873	2.3535	15.8797	0.0009	0.0286
0.4624	2.8334	20.6197	0.0003	0.0156
1.3460	3.4650	50.0021	0.0000	0.0013
-0.5015	2.1968	25.9154	0.0001	0.0082
0.3170	3.6675	24.9161	0.0001	0.0086
0.4643	3.9560	42.7186	0.0000	0.0018
0.4624	2.8334	20.6197	0.0003	0.0156
0.4904	2.2767	25.7504	0.0001	0.0083
-0.5015	2.1968	25.9154	0.0001	0.0082
-0.7318	1.5868	12.6570	0.0023	0.0458
0.6093	5.5716	23.8912	0.0001	0.0099
0.3211	7.1475	12.8832	0.0021	0.0437
0.3278	3.1712	17.1394	0.0006	0.0242
1.3460	3.4650	50.0021	0.0000	0.0013
0.6381	5.0308	14.1739	0.0015	0.0371
0.3577	2.6804	18.6195	0.0004	0.0197
0.6891	4.0716	13.4865	0.0019	0.0413
1.3460	3.4650	50.0021	0.0000	0.0013
0.3912	3.3627	20.7318	0.0003	0.0153
0.4258	7.3496	26.8295	0.0001	0.0075
1.0351	6.7272	15.2373	0.0012	0.0331
0.6093	5.5716	23.8912	0.0001	0.0099
0.6891	4.0716	13.4865	0.0019	0.0413
0.6269	2.3660	15.0734	0.0011	0.0316
0.2700	7.9524	20.2412	0.0003	0.0164
2.2188	4.8714	21.2799	0.0003	0.0181
2.0169	1.3312	15.5315	0.0012	0.0323

1.3908	0.3673	19.2529	0.0004	0.0192
0.3327	5.1894	17.5313	0.0006	0.0229
0.8186	-0.0911	25.2102	0.0001	0.0080
-0.4112	2.6413	13.0801	0.0020	0.0424
0.3577	2.6804	18.6195	0.0004	0.0197
0.5569	6.6421	20.2848	0.0003	0.0163
0.2391	4.3802	12.5866	0.0023	0.0464
-0.3451	4.7455	14.7714	0.0012	0.0330
-0.7668	6.0634	47.6969	0.0000	0.0014
2.0870	3.2489	39.1226	0.0000	0.0034
0.7415	4.0844	13.6770	0.0018	0.0404
1.3460	3.4650	50.0021	0.0000	0.0013
3.9549	2.7136	20.3619	0.0002	0.0135
-0.3176	5.9710	12.8947	0.0021	0.0437
0.4624	2.8334	20.6197	0.0003	0.0156
0.4643	3.9560	42.7186	0.0000	0.0018
0.3577	2.6804	18.6195	0.0004	0.0197
-0.3451	4.7455	14.7714	0.0012	0.0330
-0.2794	6.7890	13.4386	0.0018	0.0407
-0.3873	2.3535	15.8797	0.0009	0.0286
-0.6625	7.7608	25.5578	0.0001	0.0083
-0.5015	2.1968	25.9154	0.0001	0.0082
0.3612	8.0741	14.5054	0.0013	0.0341
0.5328	5.3797	17.0026	0.0006	0.0247
0.3658	6.0270	16.0492	0.0008	0.0280
0.2617	3.1756	15.5926	0.0010	0.0297
-0.7318	1.5868	12.6570	0.0023	0.0458
-0.2373	5.3333	13.9044	0.0016	0.0379
1.0351	6.7272	15.2373	0.0012	0.0331
1.3460	3.4650	50.0021	0.0000	0.0013
-0.7566	3.7974	76.6720	0.0000	0.0003
1.3460	3.4650	50.0021	0.0000	0.0013
0.5975	2.4916	26.6277	0.0001	0.0076
0.7791	1.7234	17.8251	0.0005	0.0222
0.3414	5.2054	18.7713	0.0004	0.0196
4.1234	5.1954	28.8053	0.0001	0.0082
0.7867	2.8742	15.6469	0.0010	0.0299
3.6753	4.5531	16.7786	0.0010	0.0299
0.6093	5.5716	23.8912	0.0001	0.0099
-0.2919	3.6510	20.7294	0.0003	0.0153
1.3460	3.4650	50.0021	0.0000	0.0013
4.1234	5.1954	28.8053	0.0001	0.0082
-0.3873	2.3535	15.8797	0.0009	0.0286
0.2915	5.9320	22.7570	0.0002	0.0117
0.4087	5.6475	26.2513	0.0001	0.0080
0.2982	3.9210	13.3905	0.0018	0.0410
1.3460	3.4650	50.0021	0.0000	0.0013
0.3577	2.6804	18.6195	0.0004	0.0197
0.2926	6.1826	13.7604	0.0016	0.0388
0.2893	5.5537	15.6256	0.0009	0.0296
0.5332	3.5605	17.9491	0.0005	0.0218
1.3460	3.4650	50.0021	0.0000	0.0013
0.5975	2.4916	26.6277	0.0001	0.0076
0.7791	1.7234	17.8251	0.0005	0.0222
0.3577	2.6804	18.6195	0.0004	0.0197
0.3414	5.2054	18.7713	0.0004	0.0196
-0.7318	1.5868	12.6570	0.0023	0.0458
1.6279	-0.2524	35.9301	0.0000	0.0033
3.9581	2.7826	29.8769	0.0000	0.0044

3.6753	4.5531	16.7786	0.0010	0.0299
1.3460	3.4650	50.0021	0.0000	0.0013
0.3912	3.3627	20.7318	0.0003	0.0153
-0.3873	2.3535	15.8797	0.0009	0.0286
-0.4567	3.5603	13.9638	0.0015	0.0375
-0.7668	6.0634	47.6969	0.0000	0.0014
1.3460	3.4650	50.0021	0.0000	0.0013
0.6093	5.5716	23.8912	0.0001	0.0099
0.4087	5.6475	26.2513	0.0001	0.0080
0.6691	3.6192	22.9722	0.0001	0.0114
-0.4567	3.5603	13.9638	0.0015	0.0375
-0.3176	5.9710	12.8947	0.0021	0.0437
-0.3873	2.3535	15.8797	0.0009	0.0286
1.3460	3.4650	50.0021	0.0000	0.0013
1.1673	1.9510	13.7783	0.0017	0.0399
0.7867	2.8742	15.6469	0.0010	0.0299
1.2364	2.8412	23.6961	0.0001	0.0112
0.7084	2.0394	16.1266	0.0008	0.0278
-0.6897	1.0753	15.7824	0.0009	0.0291
-0.2608	7.2955	14.4879	0.0013	0.0341
-0.2919	3.6510	20.7294	0.0003	0.0153
-0.3873	2.3535	15.8797	0.0009	0.0286
0.6269	2.3660	15.0734	0.0011	0.0316
0.4572	1.5029	16.5532	0.0007	0.0259
1.2762	1.6610	22.8294	0.0002	0.0117
0.3177	4.7516	14.6575	0.0012	0.0332
0.3577	2.6804	18.6195	0.0004	0.0197
0.2768	4.1864	16.0130	0.0008	0.0281
-0.3176	5.9710	12.8947	0.0021	0.0437
-0.4659	2.4245	13.3557	0.0018	0.0413
-0.4825	4.6315	70.2957	0.0000	0.0004
-0.6272	5.3077	18.5564	0.0004	0.0200
-0.4999	3.4671	18.9264	0.0004	0.0195
1.3460	3.4650	50.0021	0.0000	0.0013
4.1234	5.1954	28.8053	0.0001	0.0082
0.6381	5.0308	14.1739	0.0015	0.0371
-0.6463	2.5018	44.6529	0.0000	0.0016
0.2768	4.1864	16.0130	0.0008	0.0281
0.5332	3.5605	17.9491	0.0005	0.0218
0.7084	2.0394	16.1266	0.0008	0.0278
0.6070	2.3683	13.2338	0.0019	0.0418
-0.6272	5.3077	18.5564	0.0004	0.0200
0.5332	3.5605	17.9491	0.0005	0.0218
0.7084	2.0394	16.1266	0.0008	0.0278
-0.3176	5.9710	12.8947	0.0021	0.0437
0.7312	4.6298	35.5110	0.0000	0.0037
0.7069	3.1144	29.1881	0.0000	0.0056
2.3373	0.9517	27.1856	0.0000	0.0061
1.0351	6.7272	15.2373	0.0012	0.0331
0.5980	2.6344	33.6618	0.0000	0.0041
-0.4842	1.0461	16.5704	0.0007	0.0259
-0.3345	4.8626	14.2851	0.0014	0.0355
-1.0513	3.7214	29.1514	0.0000	0.0060
-0.5604	4.4847	15.0868	0.0011	0.0316
0.6093	5.5716	23.8912	0.0001	0.0099
1.6255	2.8542	14.6589	0.0015	0.0368
0.7109	3.6736	19.1794	0.0004	0.0192
-0.3873	2.3535	15.8797	0.0009	0.0286
-0.7318	1.5868	12.6570	0.0023	0.0458

1.3460	3.4650	50.0021	0.0000	0.0013
-0.4611	2.7184	15.9888	0.0009	0.0281
0.6381	5.0308	14.1739	0.0015	0.0371
1.3460	3.4650	50.0021	0.0000	0.0013
1.3460	3.4650	50.0021	0.0000	0.0013
1.0418	2.3525	14.2629	0.0015	0.0370
0.5775	2.7136	12.4464	0.0024	0.0474
-0.3873	2.3535	15.8797	0.0009	0.0286
1.3460	3.4650	50.0021	0.0000	0.0013
0.3912	3.3627	20.7318	0.0003	0.0153
1.7505	5.7691	26.5695	0.0001	0.0085
0.5697	6.3701	87.8065	0.0000	0.0002
0.2501	5.5907	17.8782	0.0005	0.0220
0.5273	5.2741	17.6575	0.0005	0.0227
-0.3873	2.3535	15.8797	0.0009	0.0286
-0.4691	4.2077	16.8934	0.0007	0.0250
0.6093	5.5716	23.8912	0.0001	0.0099
0.6169	3.7040	32.7979	0.0000	0.0041
0.9947	-0.0183	18.7395	0.0004	0.0197
1.0418	2.3525	14.2629	0.0015	0.0370
0.2887	4.2694	24.6984	0.0001	0.0087
2.5425	5.3512	19.4088	0.0005	0.0219
-0.2517	7.5526	13.5754	0.0017	0.0399
-0.3176	5.9710	12.8947	0.0021	0.0437
-0.3873	2.3535	15.8797	0.0009	0.0286
4.1234	5.1954	28.8053	0.0001	0.0082
0.2887	4.2694	24.6984	0.0001	0.0087
0.5704	7.6191	13.4888	0.0018	0.0408
0.4454	7.2762	15.3787	0.0010	0.0302
0.3912	3.3627	20.7318	0.0003	0.0153
1.3460	3.4650	50.0021	0.0000	0.0013
0.6381	5.0308	14.1739	0.0015	0.0371
1.3460	3.4650	50.0021	0.0000	0.0013
-0.3873	2.3535	15.8797	0.0009	0.0286
-0.6454	4.7202	21.6802	0.0002	0.0137
0.5332	3.5605	17.9491	0.0005	0.0218
1.3460	3.4650	50.0021	0.0000	0.0013
-0.7318	1.5868	12.6570	0.0023	0.0458
-0.2794	6.7890	13.4386	0.0018	0.0407
-0.6625	7.7608	25.5578	0.0001	0.0083
-0.3176	5.9710	12.8947	0.0021	0.0437
-0.5322	5.3994	17.8043	0.0005	0.0223
1.3460	3.4650	50.0021	0.0000	0.0013
0.2753	6.8145	14.8396	0.0012	0.0328
-0.7668	6.0634	47.6969	0.0000	0.0014
-0.7318	1.5868	12.6570	0.0023	0.0458
0.3414	5.2054	18.7713	0.0004	0.0196
0.2743	4.5520	13.3779	0.0018	0.0411
0.8878	3.4701	13.0510	0.0022	0.0447
0.5775	2.7136	12.4464	0.0024	0.0474
0.6741	4.5405	16.1855	0.0008	0.0281
1.9788	2.7562	19.8128	0.0004	0.0195
-0.5015	2.1968	25.9154	0.0001	0.0082
-0.6625	7.7608	25.5578	0.0001	0.0083
-0.6463	2.5018	44.6529	0.0000	0.0016
0.6093	5.5716	23.8912	0.0001	0.0099
0.4410	3.7555	14.5708	0.0013	0.0337
-0.2550	13.6189	5.8762	0.0262	0.1573
-0.6625	7.7608	25.5578	0.0001	0.0083

-0.3324	3.3313	17.3631	0.0006	0.0234
4.1234	5.1954	28.8053	0.0001	0.0082
0.3120	5.5347	12.7102	0.0022	0.0452
-0.3903	2.6115	13.2370	0.0019	0.0418
-0.3451	4.7455	14.7714	0.0012	0.0330
0.8320	6.1915	35.8840	0.0000	0.0035
0.3912	3.3627	20.7318	0.0003	0.0153
4.6051	4.6138	18.0442	0.0004	0.0201
-1.1916	3.5629	49.5312	0.0000	0.0013
-0.3873	2.3535	15.8797	0.0009	0.0286
-0.4567	3.5603	13.9638	0.0015	0.0375
1.3460	3.4650	50.0021	0.0000	0.0013
0.2501	5.5907	17.8782	0.0005	0.0220
0.3747	8.0026	21.5331	0.0002	0.0141
0.3984	5.5924	32.7891	0.0000	0.0041
0.7069	3.1144	29.1881	0.0000	0.0056
-0.5072	2.1776	14.4357	0.0013	0.0345
0.3482	3.8639	19.8194	0.0003	0.0174
1.6255	2.8542	14.6589	0.0015	0.0368
0.2915	5.9320	22.7570	0.0002	0.0117
0.3278	3.1712	17.1394	0.0006	0.0242
0.3369	5.0913	13.4289	0.0018	0.0407
-0.2721	5.5390	18.9009	0.0004	0.0195
-0.7318	1.5868	12.6570	0.0023	0.0458
-0.5322	5.3994	17.8043	0.0005	0.0223
0.2862	4.0537	13.1916	0.0019	0.0420
0.5569	6.6421	20.2848	0.0003	0.0163
0.2391	4.3802	12.5866	0.0023	0.0464
0.8878	3.4701	13.0510	0.0022	0.0447
0.3832	3.3573	18.8399	0.0004	0.0195
3.9581	2.7826	29.8769	0.0000	0.0044
3.6753	4.5531	16.7786	0.0010	0.0299
1.3460	3.4650	50.0021	0.0000	0.0013
0.8320	6.1915	35.8840	0.0000	0.0035
1.3908	0.3673	19.2529	0.0004	0.0192
-1.1916	3.5629	49.5312	0.0000	0.0013
1.3460	3.4650	50.0021	0.0000	0.0013
-0.3176	5.9710	12.8947	0.0021	0.0437
-0.6975	0.0317	15.8117	0.0009	0.0291
0.3912	3.3627	20.7318	0.0003	0.0153
-0.6463	2.5018	44.6529	0.0000	0.0016
1.3460	3.4650	50.0021	0.0000	0.0013
-0.3176	5.9710	12.8947	0.0021	0.0437
0.5980	2.6344	33.6618	0.0000	0.0041
0.6093	5.5716	23.8912	0.0001	0.0099
0.7109	3.6736	19.1794	0.0004	0.0192
-0.3176	5.9710	12.8947	0.0021	0.0437
1.3460	3.4650	50.0021	0.0000	0.0013
-0.2924	7.2526	14.4969	0.0013	0.0341
-0.3873	2.3535	15.8797	0.0009	0.0286
-0.4567	3.5603	13.9638	0.0015	0.0375
1.3460	3.4650	50.0021	0.0000	0.0013
0.3369	5.0913	13.4289	0.0018	0.0407
1.1543	3.7547	16.0412	0.0010	0.0299
1.1559	2.8092	13.2461	0.0021	0.0440
0.5775	2.7136	12.4464	0.0024	0.0474
0.3912	3.3627	20.7318	0.0003	0.0153
0.5317	1.8929	15.4515	0.0010	0.0299
1.3460	3.4650	50.0021	0.0000	0.0013

-0.4344	3.9918	12.7219	0.0022	0.0451
-0.4691	4.2077	16.8934	0.0007	0.0250
-0.3873	2.3535	15.8797	0.0009	0.0286
1.3460	3.4650	50.0021	0.0000	0.0013
0.5775	2.7136	12.4464	0.0024	0.0474
0.3369	5.0913	13.4289	0.0018	0.0407
0.5980	2.6344	33.6618	0.0000	0.0041
-0.6463	2.5018	44.6529	0.0000	0.0016
0.5980	2.6344	33.6618	0.0000	0.0041
0.8538	4.1507	18.4253	0.0005	0.0211
1.0418	2.3525	14.2629	0.0015	0.0370
0.8320	6.1915	35.8840	0.0000	0.0035
0.2391	4.3802	12.5866	0.0023	0.0464
-0.3873	2.3535	15.8797	0.0009	0.0286
-0.4567	3.5603	13.9638	0.0015	0.0375
-0.6463	2.5018	44.6529	0.0000	0.0016
-0.4112	2.6413	13.0801	0.0020	0.0424
1.3460	3.4650	50.0021	0.0000	0.0013
0.3369	5.0913	13.4289	0.0018	0.0407
0.5501	2.7216	14.5936	0.0013	0.0336
0.5503	6.4557	15.1611	0.0011	0.0311
-0.6054	3.7801	17.6843	0.0005	0.0227
1.3460	3.4650	50.0021	0.0000	0.0013
0.3912	3.3627	20.7318	0.0003	0.0153
-0.3903	2.6115	13.2370	0.0019	0.0418
-0.3873	2.3535	15.8797	0.0009	0.0286
0.7109	3.6736	19.1794	0.0004	0.0192
1.3460	3.4650	50.0021	0.0000	0.0013
1.0418	2.3525	14.2629	0.0015	0.0370
3.6753	4.5531	16.7786	0.0010	0.0299
-0.6463	2.5018	44.6529	0.0000	0.0016
0.2687	3.5465	13.6076	0.0017	0.0397
1.3460	3.4650	50.0021	0.0000	0.0013
0.6741	4.5405	16.1855	0.0008	0.0281
1.3460	3.4650	50.0021	0.0000	0.0013
1.1559	2.8092	13.2461	0.0021	0.0440
0.5775	2.7136	12.4464	0.0024	0.0474
-0.4691	4.2077	16.8934	0.0007	0.0250
-0.3873	2.3535	15.8797	0.0009	0.0286
0.3369	5.0913	13.4289	0.0018	0.0407
0.3912	3.3627	20.7318	0.0003	0.0153
1.3460	3.4650	50.0021	0.0000	0.0013
0.4873	4.0931	12.5508	0.0023	0.0467
-0.3458	6.1748	18.5498	0.0004	0.0198
1.3460	3.4650	50.0021	0.0000	0.0013
1.1543	3.7547	16.0412	0.0010	0.0299
0.6195	2.9529	15.3370	0.0010	0.0304
0.2887	4.2694	24.6984	0.0001	0.0087
1.3846	5.9599	21.4943	0.0003	0.0158
1.1559	2.8092	13.2461	0.0021	0.0440
0.5317	1.8929	15.4515	0.0010	0.0299
-0.4344	3.9918	12.7219	0.0022	0.0451
-0.4567	3.5603	13.9638	0.0015	0.0375
4.6051	4.6138	18.0442	0.0004	0.0201
-0.3873	2.3535	15.8797	0.0009	0.0286
3.9581	2.7826	29.8769	0.0000	0.0044
0.7415	4.0844	13.6770	0.0018	0.0404
-0.3268	2.8278	21.7668	0.0002	0.0136
1.3460	3.4650	50.0021	0.0000	0.0013

0.3912	3.3627	20.7318	0.0003	0.0153
-0.3176	5.9710	12.8947	0.0021	0.0437
1.3460	3.4650	50.0021	0.0000	0.0013
0.7069	3.1144	29.1881	0.0000	0.0056
2.1867	9.1171	15.1248	0.0014	0.0366
0.4454	7.2762	15.3787	0.0010	0.0302
0.5761	5.6917	15.2554	0.0011	0.0309
-0.7668	6.0634	47.6969	0.0000	0.0014
0.7415	4.0844	13.6770	0.0018	0.0404
1.3460	3.4650	50.0021	0.0000	0.0013
-0.3176	5.9710	12.8947	0.0021	0.0437
1.0418	2.3525	14.2629	0.0015	0.0370
1.3460	3.4650	50.0021	0.0000	0.0013
-0.4567	3.5603	13.9638	0.0015	0.0375
-0.3873	2.3535	15.8797	0.0009	0.0286
0.4873	4.0931	12.5508	0.0023	0.0467
0.7109	3.6736	19.1794	0.0004	0.0192
-0.2517	7.5526	13.5754	0.0017	0.0399
-0.3458	6.1748	18.5498	0.0004	0.0198
-0.2664	11.7779	17.5248	0.0006	0.0229
-0.2811	6.4295	20.1084	0.0003	0.0167
0.4873	4.0931	12.5508	0.0023	0.0467
0.7109	3.6736	19.1794	0.0004	0.0192
-0.2517	7.5526	13.5754	0.0017	0.0399
-0.3458	6.1748	18.5498	0.0004	0.0198
-0.2664	11.7779	17.5248	0.0006	0.0229
-0.2811	6.4295	20.1084	0.0003	0.0167
0.4873	4.0931	12.5508	0.0023	0.0467
1.0372	2.8591	17.1756	0.0007	0.0250
-0.2517	7.5526	13.5754	0.0017	0.0399
-0.3458	6.1748	18.5498	0.0004	0.0198
-0.2811	6.4295	20.1084	0.0003	0.0167
0.3369	5.0913	13.4289	0.0018	0.0407
0.4873	4.0931	12.5508	0.0023	0.0467
1.3460	3.4650	50.0021	0.0000	0.0013
3.6753	4.5531	16.7786	0.0010	0.0299
0.3912	3.3627	20.7318	0.0003	0.0153
-0.3176	5.9710	12.8947	0.0021	0.0437
-0.4691	4.2077	16.8934	0.0007	0.0250
1.0372	2.8591	17.1756	0.0007	0.0250
-0.3873	2.3535	15.8797	0.0009	0.0286
-0.6625	7.7608	25.5578	0.0001	0.0083
1.3460	3.4650	50.0021	0.0000	0.0013
0.3369	5.0913	13.4289	0.0018	0.0407
0.5980	2.6344	33.6618	0.0000	0.0041
-0.4691	4.2077	16.8934	0.0007	0.0250
0.3327	5.1894	17.5313	0.0006	0.0229
0.6195	2.9529	15.3370	0.0010	0.0304
-0.5322	5.3994	17.8043	0.0005	0.0223
-0.2811	6.4295	20.1084	0.0003	0.0167
0.3912	3.3627	20.7318	0.0003	0.0153
-0.3873	2.3535	15.8797	0.0009	0.0286
0.4881	5.5673	19.3232	0.0004	0.0190
0.6181	7.0850	21.4849	0.0002	0.0142
0.3218	5.2160	13.0157	0.0020	0.0427
0.2847	2.7978	14.7981	0.0012	0.0329
4.6051	4.6138	18.0442	0.0004	0.0201
3.9581	2.7826	29.8769	0.0000	0.0044
3.6753	4.5531	16.7786	0.0010	0.0299

0.4873	4.0931	12.5508	0.0023	0.0467
-0.3873	2.3535	15.8797	0.0009	0.0286
0.4190	5.7579	34.0132	0.0000	0.0041
0.5363	2.6703	21.1012	0.0002	0.0149
-0.4485	1.9227	15.4621	0.0010	0.0299
0.3395	3.2347	25.5326	0.0001	0.0083
-0.2762	4.4094	21.7239	0.0002	0.0137
0.3519	6.5941	27.3885	0.0001	0.0070
1.7505	5.7691	26.5695	0.0001	0.0085
0.5697	6.3701	87.8065	0.0000	0.0002
-0.3873	2.3535	15.8797	0.0009	0.0286
-0.4567	3.5603	13.9638	0.0015	0.0375
-0.4429	5.5858	15.6551	0.0009	0.0296
-0.4567	3.5603	13.9638	0.0015	0.0375
-0.3873	2.3535	15.8797	0.0009	0.0286
-0.9123	5.2655	75.5953	0.0000	0.0003
-0.7418	4.1843	17.2233	0.0007	0.0247
0.7069	3.1144	29.1881	0.0000	0.0056
0.3747	8.0026	21.5331	0.0002	0.0141
0.3984	5.5924	32.7891	0.0000	0.0041
0.2501	5.5907	17.8782	0.0005	0.0220
-0.6454	4.7202	21.6802	0.0002	0.0137
-0.5072	2.1776	14.4357	0.0013	0.0345
0.7109	3.6736	19.1794	0.0004	0.0192
0.2887	4.2694	24.6984	0.0001	0.0087
0.3912	3.3627	20.7318	0.0003	0.0153
-0.3873	2.3535	15.8797	0.0009	0.0286
-0.4567	3.5603	13.9638	0.0015	0.0375
-0.4691	4.2077	16.8934	0.0007	0.0250
0.6381	5.0308	14.1739	0.0015	0.0371
0.3047	3.1190	14.1844	0.0014	0.0363
-0.6731	5.0064	34.3066	0.0000	0.0041
-0.2550	13.6189	5.8762	0.0262	0.1573
-0.2550	13.6189	5.8762	0.0262	0.1573
-1.0513	3.7214	29.1514	0.0000	0.0060
0.7069	3.1144	29.1881	0.0000	0.0056
0.4454	7.2762	15.3787	0.0010	0.0302
-0.3176	5.9710	12.8947	0.0021	0.0437
-0.2517	7.5526	13.5754	0.0017	0.0399
-0.2664	11.7779	17.5248	0.0006	0.0229
0.3747	8.0026	21.5331	0.0002	0.0141
0.3984	5.5924	32.7891	0.0000	0.0041
0.2501	5.5907	17.8782	0.0005	0.0220
0.7069	3.1144	29.1881	0.0000	0.0056
-0.6731	5.0064	34.3066	0.0000	0.0041
-0.5072	2.1776	14.4357	0.0013	0.0345
-0.4400	5.6372	19.9640	0.0003	0.0169
-0.3873	2.3535	15.8797	0.0009	0.0286
-0.4567	3.5603	13.9638	0.0015	0.0375
-0.3176	5.9710	12.8947	0.0021	0.0437
1.7061	2.2893	15.2344	0.0012	0.0331
1.6255	2.8542	14.6589	0.0015	0.0368
-0.6463	2.5018	44.6529	0.0000	0.0016
0.4454	7.2762	15.3787	0.0010	0.0302
0.5761	5.6917	15.2554	0.0011	0.0309
0.2879	4.4589	18.8239	0.0004	0.0195
0.2501	5.5907	17.8782	0.0005	0.0220
0.2887	4.2694	24.6984	0.0001	0.0087
-0.3873	2.3535	15.8797	0.0009	0.0286

-0.4567	3.5603	13.9638	0.0015	0.0375
-0.3176	5.9710	12.8947	0.0021	0.0437
